# Surface Plasmonic Sensors: Sensing Mechanism and Recent Applications

**DOI:** 10.3390/s21165262

**Published:** 2021-08-04

**Authors:** Qilin Duan, Yineng Liu, Shanshan Chang, Huanyang Chen, Jin-hui Chen

**Affiliations:** 1 Institute of Electromagnetics and Acoustics and Key Laboratory of Electromagnetic Wave Science and Detection Technology, Xiamen University, Xiamen 361005, China; duanqilin@stu.xmu.edu.cn (Q.D.); lyn610@xmu.edu.cn (Y.L.); changshanshan@stu.xmu.edu.cn (S.C.); 2 School of Electronic Science and Engineering, Xiamen University, Xiamen 361005, China; 3 Shenzhen Research Institute, Xiamen University, Shenzhen 518000, China

**Keywords:** surface plasmonic sensors, metastructure, optical fiber, quantum plasmonic sensors

## Abstract

Surface plasmonic sensors have been widely used in biology, chemistry, and environment monitoring. These sensors exhibit extraordinary sensitivity based on surface plasmon resonance (SPR) or localized surface plasmon resonance (LSPR) effects, and they have found commercial applications. In this review, we present recent progress in the field of surface plasmonic sensors, mainly in the configurations of planar metastructures and optical-fiber waveguides. In the metastructure platform, the optical sensors based on LSPR, hyperbolic dispersion, Fano resonance, and two-dimensional (2D) materials integration are introduced. The optical-fiber sensors integrated with LSPR/SPR structures and 2D materials are summarized. We also introduce the recent advances in quantum plasmonic sensing beyond the classical shot noise limit. The challenges and opportunities in this field are discussed.

## 1. Introduction

In the last century, numerous pioneering researchers have laid the foundations of plasmonic optics [1,2,3,4,5,6]. This has stimulated significant advances in negative refractive index materials, optical metasurfaces, and integrated circuits, etc. [7,8,9,10,11,12]. Surface plasmon resonance (SPR) is the resonant coupling of electromagnetic waves to the charge density oscillations at the interface of dielectrics and metals, which is also recognized as surface plasmon polaritons (SPPs) [13]. Given the large optical momentum mismatch between SPP-mode and light in free space, the optical excitations in SPR are usually realized by the method of attenuated total reflection, proposed by Kretschmann [14] and Otto [15]. The optical gratings [16,17] and waveguides with compact coupling structures [18] are also widely employed for SPR-integrated circuits. The localized surface plasmon resonance (LSPR) is collective electron oscillation in metal nanoparticles coupled with an electromagnetic field [7]. From the optical mode analysis, the SPR modes are bound solutions to the wave equation, while LSPR are responsive modes to the illuminated light field, which is highly damped [19]. Thus, the LSPR can be directly excited from free space without following the critical momentum matching.

**Figure 1:**
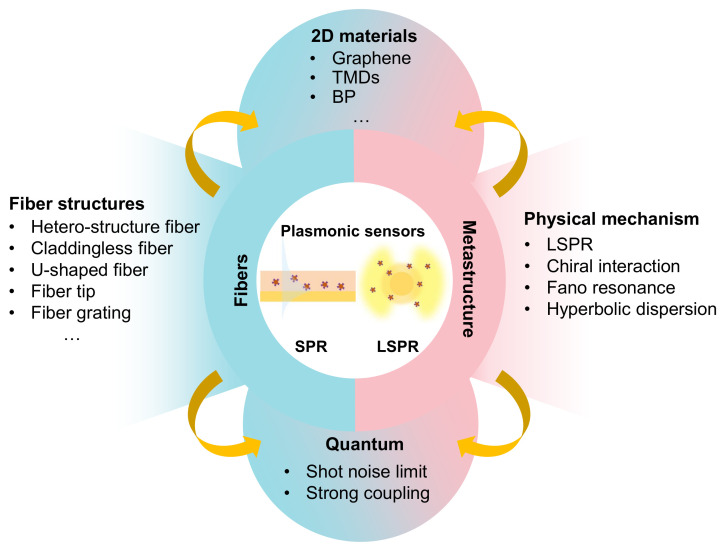
An overview of this review, the center is the sensing mechanism of plasmonic sensors.

Both SPR and LSPR structures are attracting platforms for the optical sensors due to their strong confinement and enhancement of the electromagnetic field near the surface [20,21]. The representative diagrams of SPR and LSPR used for sensing are shown in the center of Figure 1. The typical decay length of SPR structure is of 100–600 nm [22], and the LSPR has much shorter decay length of 5–60 nm [23]. Thus, it is regarded that the SPR structure is more suitable for the detection of larger biomolecules, while LSPR is better for tracing smaller biomolecules. Optical plasmonic sensors have already exhibited the ability of real time, label-free sensing, and have shown extraordinary sensitivity. They have had successful applications in the field of chemistry [24], biology [25,26,27], medicine [28] and food safety [29], etc. In general, the mechanism of these sensors is mainly based on the single mode of SPR and LSPR or their hybrid modes. Theoretically, SPR and LSPR have the advantages of tunable resonant response in wavelength, intensity and phase, and the plasmonic sensors can be optimized through manipulating the geometric structures and functional materials.

The light-matter interaction through the evanescent field of plasmonic structures is the common mechanism for analytes detection [30]. The detecting approaches used in SPR/LSPR sensors include analyzing the changes of wavelength [31], angular [32], phase [33] and polarization [34] parameters of the plasmonic structures. Note that the basic physical parameter that can be directly measured is light intensity; thus, different interrogation systems conventionally utilize optical instruments to convert changes in phase, angle, polarization into intensity variations [35]. Despite the tremendous success of plasmonic sensors, it is recognized that the sensitivity and precision of plasmonic sensors are beginning to reach a fundamental limit by quantum fluctuations of light known as shot-noise limit (SNL) regardless of whether intensity or phase is measured [36,37]. Recently, quantum techniques have been identified to out-perform classical sensing methods and achieve sensitivity beyond the SNL [38,39,40,41,42].

In this review, we mainly introduce the recent progress of plasmonic refractive index (RI) sensors from classical measurement to the quantum metrology as summarized in Figure 1. In Section 2, we discuss the basic figure of merits to evaluate plasmonic sensors, which can be readily extended to other optical sensors. In Section 3, we mainly focus on metastructure-based plasmonic sensors with different physical effects and functional materials. In particular, we introduce the LSPR-based RI sensing, and the emerging field of chiral sensors are highlighted. Hyperbolic metamaterials with indefinite in-plane momentum and Fano resonances with asymmetric sharp spectra are discussed for sensing applications. In Section 4, the LSPR and SPR modes in optical fiber-based plasmonic sensors are introduced. The integration of two-dimensional (2D) materials with excellent physical and chemical properties in plasmonics structures are reviewed. In Section 5, we introduce the rising field of quantum plasmonic sensing. The plasmonic sensors with quantum state measurement and cavity quantum electrodynamics are introduced. Finally we discussed the challenges and opportunities in plasmonic sensors.

## 2. Performance Characteristics of Plasmonic Sensors

In this section, we discuss the basic parameters that are used to evaluate the plasmonic RI-sensors. The bulk RI sensitivity of optical sensors is generally defined as [30]:

(1) $$S=\frac{dA}{dn}$$

where *A* represents the parameters that are measured (angular, wavelength or intensity), and *n* is the RI of analytes. For plasmonic sensors, the *S* can be varied from 50 nm/RIU (refractive index unit) to 30,000 nm/RIU depending on the designed structures and materials [43]. In addition, the linear relationship between *A* and *n* is usually required for practical applications, so a correlation coefficient factor can also be taken into consideration [44].

Another important parameter is figure of merit (FOM) [45], which is defined as:

(2) $$\mathrm{FOM}=\frac{S}{\mathrm{FWHM}}$$

where the FWHM is the full width at half maximum of resonance spectra. The FOM defines the ability of sensors to measure small RI change; thus, a larger FOM is desirable. Generally, the FOM of plasmonic sensors is low due to the intrinsic loss of metals. Fortunately, by leveraging the optical field coupling effects or the chiral interactions, the FOM can be significantly improved as will be discussed in Section 3.

The other parameter to describe the performance of plasmonic sensors is limit of detection (LOD), which resolves the smallest quantity of change that can be detected by the system. LOD can be defined as [46,47]:

(3) $$\mathrm{LOD}=m\frac{\sigma_{\mathrm{blank}}\;}{S}$$

where m is a numerical factor (typically 2 or 3), $\sigma_{\mathrm{blank}\;}$ is the standard deviation of the blank measures. Since LOD is determined by both the sensitivity and the noise level, it can be improved by using less-noise detectors and sources. Recently, Jin et al. demonstrated an up-converted detection system which can suppress sampling noise amplitude by two orders of magnitude [48]. The ultralow-noise sensor is applied to detect the single polystyrene particle with the detection limit down to a few attogram. Thus, improving the measurement method is an alternative way to reduce the LOD. Quantum optics applied in sensing is another powerful technique to overcome the detection noise [41,49], which will be discussed in Section 5.

Other performance characteristics such as accuracy, reproducibility and dynamic range are also important parameters for optical sensors [46]. The sensor accuracy describes the closeness between a measured value and a true value. Reproducibility is the ability to provide the stable output under the same operating conditions. The dynamic range describes the range of values that can be measured by the sensors. We note that for practical applications, there always exist the compromise of performance and cost of plasmonics sensors.

## 3. Metastructure-Based Plasmonic Sensors

Optical metasturctures are composed of designed artificial meta-atoms, which can manipulate the amplitude, phase and polarization of electromagnetic waves [50,51,52,53,54,55,56]. In the past years, the researches of optical metastructures have transited from bulk metamaterials to planar metasurfaces and even the curved metastructures [57,58,59,60]. For optical sensors, plasmonic metasurfaces with high quality (Q) factor resonances have been proposed by leveraging near- and far-field coupling of nanostructures. The collective mode resonance effect significantly breaks the damping limit of a single metal nanostructure under the dipole approximation.

### 3.1. LSPR-Based Metastructure Sensors

LSPR is collective oscillations of electrons at the surface of metallic nanostructures. As a typical example, consider a single spherical metal nanoparticle with radius of *a*, which is illuminated by electromagnetic field under the condition of a≪λ. By solving the Laplace equation with a boundary condition, the resonance of LSPR in a spherical nanoparticle is written as [61]:

(4) Re(εm)εd+l+1l=0

where $\varepsilon_{m}$ and $\varepsilon_{d}$ are the permittivity of metal and environment, respectively; *l* is the angular momentum of the resonant mode. In theoretical analysis, we have the Drude model of metals:

(5) $$\varepsilon_{m}\left(\omega \right)=1-\frac{\omega_{p}^{2}}{\omega^{2}+i\gamma \omega }$$

where $\omega_{p}$ and γ are the plasma frequency and damping rate of metals, respectively. Substitute Equation (5) into Equation (4) and ignore the damping effect, the resonance frequency of LSP is derived:

(6) $$\omega_{l}=\omega_{p}{\left[\frac{l}{\varepsilon_{d}\left(l+1\right)+l}\right]}^{1/2}$$

**Figure 2:**
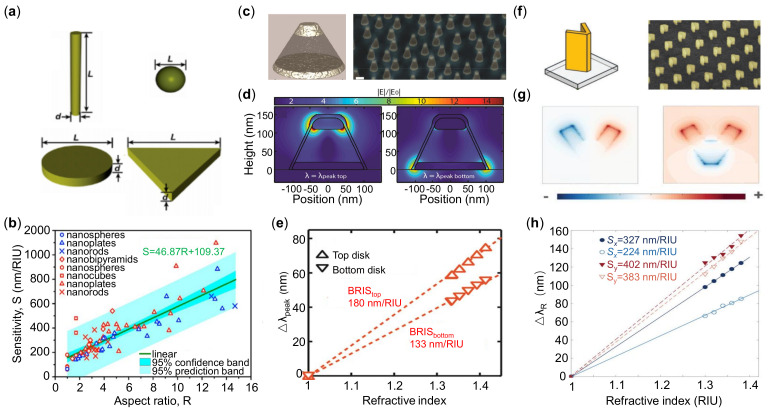
Localized surface plasmon resonance (LSPR) structures and sensing performance. (**a**) Various shapes of plasmonic nanoparticles. (**b**) Refractive index (RI) sensitivity of plasmonic nanoparticles of various sizes, shapes, and compositions. (**c**) Schematic (left) and scanning electron microscope (SEM) image (right) of the 3D nanoplasmonic sensor architecture. Two Ag nanodisks of different diameters and thickness are vertically separated by a thick SiO${}_{2}$ spacer layer. (**d**) Electromagnetic field distribution by a normal-incidence plane wave at different resonance wavelengths. (**e**) Bulk RI sensitivity for top disk and bottom disk. (**f**) Schematic (left) and SEM image (right) of V-shaped antenna. (**g**) Transverse surface charge distribution. (**h**) Bulk sensitivity properties for different optical polarized light excitation in (**f**). Reprinted (**a**,**b**) with permission from Reference [63]. Reprinted (**c**–**e**) with permission from Reference [64]. Reprinted (**f**–**h**) with permission from Reference [65].

The variation of background permittivity $\varepsilon_{d}$ will lead to the spectral shift Δω (correspondingly Δλ for wavelength) of LSPR, which is the basic sensing mechanism [62]. Recently, Khan et al. [63] systematically measured and analyzed the sensitivities of a group of LSPR structures (Figure 2a). They found that the aspect ratio of various nanoparticles had strong correlations to the sensitivity as shown in Figure 2b. This comprehensive study can be served as a guideline for nanoparticle synthesis and fabrication of highly sensitive nanomaterials.

For the past decades, LSPR of metallic nanoparticles and their applications have been studied extensively, and there are many comprehensive review papers [13,66,67,68,69,70,71]. The LSPR with metastructures offers a strongly confined and enhanced electromagnetic field with the advantages of spectral tunability, which have been widely used in sensing applications [72,73,74]. To date, most of LSPR sensors consist of two-dimensional (2D) nanoparticle arrays, while sensing in three dimensions (3D) has huge potentials to be used for measuring the physical or chemical gradient of the analytes. A plasmonic metasurface comprising quasi-random arrays of stacked Ag nanodisks with a thick SiO${}_{2}$ intercalation layer is shown in Figure 2c [64]. Through rational design, it exhibits two distinct LSPR peaks with various sensitivity, and they correspond to spatially separated sensing locations along the axial direction as shown in Figure 2d,e.

The LSPRs of metal nanostructures generally have small FOM values due to the strong radiative damping, which significantly constrains the performances of LSPR sensors [75]. The trend in the design of LSPR sensors is to use hybrid modes, for example, LSPR -SPR, to improve the sensitivity and FOM simultaneously. In Figure 2f, the “V” shape particle lattices are explored to support a hybrid mode by the transverse LSP (Figure 2g (left)) and longitudinal Fabry–Perot modes (Figure 2g (right)) [65]. The strong polarization-dependent sensing performance of the hybrid mode is shown in Figure 2h, in which a higher sensitivity of 402 nm/RIU is achieved at longer wavelength resonance.

The detection of chiral molecules is of great importance in the field of both biology and chemistry because the enantiomers may exhibit completely different activities [76,77]. Chirality refers to structures with broken mirror symmetry such as the enantiomers, which universally exists in chemical molecules [78]. Since chiral molecules respond differently to left- and right-circular polarized light, various spectroscopic techniques have been developed to differentiate enantiomers including circular dichroism (CD), optical rotatory dispersion, and Raman optical activity [79]. However, there are some difficulties in chiral sensing such as weak chiroptical signals, and most of the significant chiroptical signals are located in the ultraviolet (UV) range (150–300 nm) [80,81]. Plasmonic metastructures show great potentials in chiral sensing for their advantages in flexibly controlling the light-matter interactions. For example, the chiral plasmonic structures can significantly enhance the CD signal of molecules and extend the CD spectra from UV into the visible regime, which simplifies the detection system [82]. Different chiral structures have been proposed for chiral molecule sensing, such as the gammadion [83,84] and shuriken [85] shape. The 3D chiral [86,87] nanostructures and twisted optical metamaterials [88] have also been reported. Hendry et al. [83] demonstrated a superchiral electromagnetic field generated by a planar plasmonic chiral metastructure. They showed that the effective refractive index of chiral molecules exposed to left- and right-handed superchiral fields are $10^{6}$ times larger than the conventional polarimetric spectroscopy.

**Figure 3:**
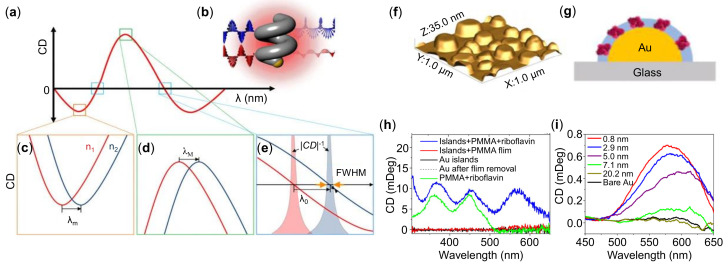
Plasmonic chiral sensors. (**a**) Circular dichroism (CD) spectra of a plasmonic enantiomer. (**b**) Illustration shows the interaction between a left-handed nanohelix and circularly polarized light. Three bottom panels indicate the resonance shifts at (**c**–**e**) where the RI of the surroundings are varied from $n_{1}$ and $n_{2}$. (**f**) Atomic force microscope (AFM) images of gold-island films. (**g**) Scheme of gold islands covered with polymers which includes embedded riboflavin molecules. (**h**) CD spectra for different hybrid plasmonic structures. (**i**) CD spectra from Riboflavin derivative with different separation from the gold surface, together with a bare gold CD spectrum. Reprinted (**a**–**e**) with permission from Reference [91]. Reprinted (**f**–**i**) with permission from Reference [92].

Using chiral plasmonic metastructures as RI sensors has some advantages compared with achiral structures, since the CD signal is robust against background interference. Wu et al. designed moire chiral metamaterials consisting of two layers of identical Au nanohole arrays [89]. In this structure, spacer-dependent near-field coupling is revealed in theory and experiment. An ultrahigh sensitivity of >10${}^{5}$ nm/RIU and a FOM of 10${}^{5}$ /RIU was measured through CD spectra. Palermo et al. [90] theoretically studied a chiral metasurface and hyperbolic metamaterial enabling both high sensitivity and specificity for molecules sensing. They argued that the helicoidal metasurface could excite surface and bulk plasmon polaritons, reduce the diffusion limit and increase the sensing surface. Besides, the CD and chiral selectivity are appropriate for biorecognition assay [90]. Figure 3a–e show the chiral particles used for LSPR sensors [91]. By employing dispersion and shape engineering of chiral particles, remarkable RI sensitivities of 1091 nm/RIU and FOM > 2800/RIU are achieved.

Recently, exploring the achiral plasmonic structures for chiral molecular sensing are developing rapidly. Theoretically, by leveraging the near-field coupling and phase retardation effects, the chiral molecules can induce CD response in the novel-metal nanostructures at the LSPR excitations [82]. Maoz et al. [92] proposed an achiral LSPR nanostructured films for chirality sensors as shown in Figure 3f,g. The decoulped nanoparticles with chiral molecules decoration were fabricated by dissolving the riboflavin (natural chiral molecule) in the PMMA solution and then coating on the Au islands. The authors systematically studied various configurations of Au island and chiral molecules as shown in Figure 3h,i. By measuring the distance dependence of the CD spectra, they found that the plasmonic CD signal was strong even for small quantity of chiral molecules at sub-10 nm separation distances.

### 3.2. Hyperbolic Dispersion Metamaterials for Sensors

**Figure 4:**
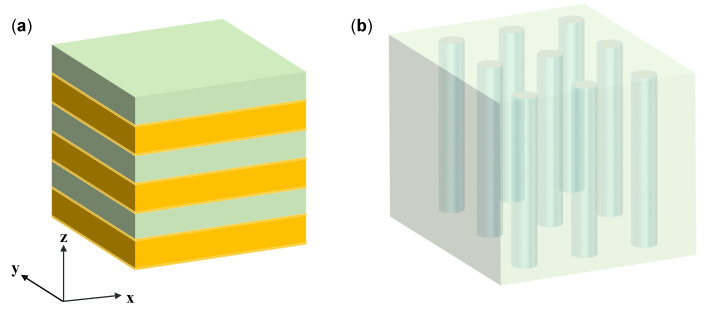
Two typical structures of hyperbolic metamaterials (HMMs). (**a**) Multilayer structure with alternating metallic and dielectric layers. (**b**) Nanowire structure consisting of metallic nanorods embedded in a dielectric host.

Hyperbolic metamaterials [93] (HMMs) are a class of metamaterials which exhibit hyperbolic dispersion with strong anisotropy. HMMs have been used for subwavelength imaging [94], and spontaneous emission enhancement [95]. Their dielectric responses are described by a diagonal tensor $\varepsilon =\left[\varepsilon_{x},\varepsilon_{y},\varepsilon_{z}\right]$, in which one component has the opposite sign to the other two. Thus, the HMMs can support waves with infinitely large momentum and strong field constraints. The HMMs are often constructed by periodically stacked materials with different permittivity (metal and dielectric material) or anisotropic nanowires [96]. The effective permittivity tensors can be deduced from Maxwell-Garnett effective medium theory (EMT). For the multilayered structure in Figure 4a, the parallel component $\left(\varepsilon_{\|}=\varepsilon_{x}=\varepsilon_{y}\right)$ and perpendicular component $\left(\varepsilon_{⊥}=\varepsilon_{z}\right)$ are given as follows [97]:

(7) $$\varepsilon_{\|}=\rho \varepsilon_{m}+\left(1-\rho \right)\varepsilon_{d}$$

(8) $$\varepsilon_{⊥}=\frac{\varepsilon_{m}\varepsilon_{d}}{\rho \varepsilon_{d}+\left(1-\rho \right)\varepsilon_{m}}$$

where $\varepsilon_{m}$ and $\varepsilon_{d}$ denote the permittivity of metal and dielectric, respectively, ρ is the filling ratio of metal. For the nanowire structure in Figure 4b, the parallel component $\left(\varepsilon_{\|}=\varepsilon_{x}=\varepsilon_{y}\right)$ and perpendicular component $\left(\varepsilon_{⊥}=\varepsilon_{z}\right)$ are derived as [97]:

(9) $$\varepsilon_{\|}=\frac{\left(1+\rho \right)\varepsilon_{m}\varepsilon_{d}+\left(1-\rho \right)\varepsilon_{d}^{2}}{\left(1+\rho \right)\varepsilon_{d}+\left(1-\rho \right)\varepsilon_{m}}$$

(10) $$\varepsilon_{⊥}=\rho \varepsilon_{m}+\left(1-\rho \right)\varepsilon_{d}$$

In plasmonic structures, the nanorod metamaterials based sensors show an enhanced sensitivity of more than 30,000 nm/RIU [98], and this structure can excite SPR as a smooth metal film with advantages of tunable spectral response and strong field localization. The excitation of high-*k* modes in a planar HMM using a grating-coupling technique [99] is an attracting platform. Sreekanth et al. [31] proposed HMM sensors integrated with a microfluidic channel, and the measured sensitivity was 30,000 nm/RIU with FOM of 590 /RIU.

**Figure 5:**
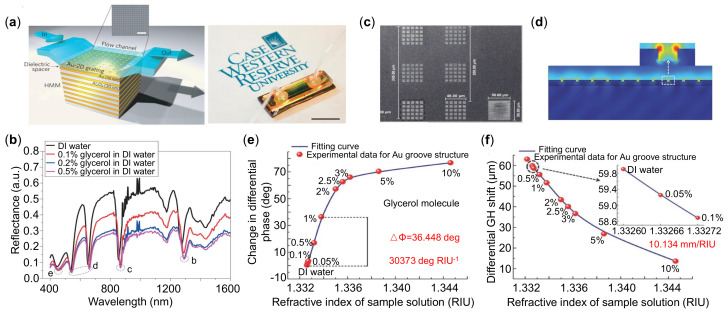
HMM-based plasmonic sensors. (**a**) Schematic of the miniaturized HMM sensing device (**left**). Photograph of the fabricated sensors (**right**). (**b**) Measured reflectance spectra of the HMM device obtained by different concentrations of glycerol. (**c**) SEM images of the nanogroove hyperbolic metasurface. (**d**) Resonant electric field distribution on the nanogroove surface. (**e**) Differential phase change of the reflected light related to the concentrations of glycerol solution. (**f**) Differential Goos–Hänchen (GH) shift with respect to the concentration of glycerol solution. Reprinted (**a**,**b**) with permission from Reference [31]. Reprinted (**c**–**f**) with permission from Reference [100].

This miniaturized sensors are promising for next-generation biosensors with high-throughput, label-free, and multi-analyte functions. In Figure 5c,d, a hyperbolic metasurface with the nanogroove structure can not only control the propagation of SPR, but also enhance light-matter interactions for sensing [100]. The strong confinement of electric field distribution is resulted from the larger effective permittivity of the plasmon mode on the ridge of the nanogroove. Thus, an ultrahigh phase sensitivity of 30,373 deg/RIU and Goos–Hänchen (GH) shift (higher order derivation of phase) sensitivity of 10.134 mm/RIU were observed as indicated in Figure 5e,f.

### 3.3. Plasmonic Sensors with Fano Resonances

Fano resonance was first proposed by Ugo Fano in 1961 [101], which is a quantum interference effect between a discrete state and a continuum. Fano resonance exhibits asymmetric spectra shape as follows [102]:

(11) $$I\propto \frac{{\left(F\gamma +\omega +\omega_{0}\right)}^{2}}{{\left(\omega -\omega_{0}\right)}^{2}+\gamma^{2}}$$

where $\omega_{0}$ and γ are the resonance frequency and linewidth, respectively; *F* is the Fano parameter that describes the degree of asymmetry. Over the past decades, Fano resonance has been realized in many optical systems containing the interference of the broad radiant and narrow subradiant modes [103,104,105,106,107]. Based on Fano resonance, a sharp asymmetric profile with small linewidth and high spectral contrast can be constructed, which has been widely used for optical sensors [108].

**Figure 6:**
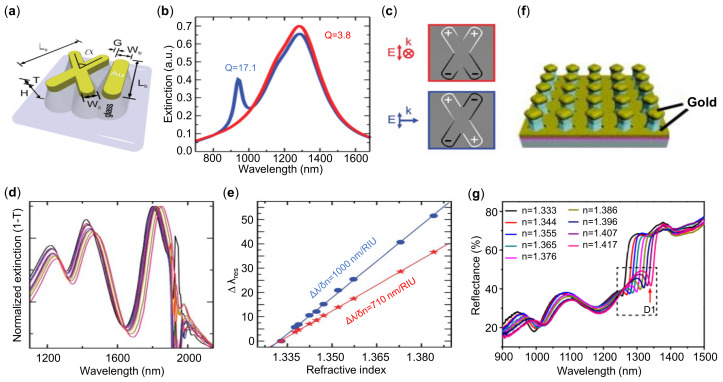
Plasmonic sensors based on Fano resonances. (**a**) Schematic of the nanocross cavity. (**b**) Calculated extinction spectra for a nanocross structure illuminated by a vertically polarized plane wave from normal (red curve) and grazing (blue curve) incidence. (**c**) Calculated charge density distribution of the dipole (top) and quadrupole mode (bottom). (**d**) RI sensing spectral response for the XI-cavity. (**e**) Spectral shift depending on the surrounding liquid for the subradiant bonding dipole-dipole mode (blue curve) and bonding quadrupole-dipole Fano resonance (red curve), respectively. (**f**) Schematic of array gold mushroom structures. (**g**) Reflectance spectra of the structure from (**f**) immersed in glycerine water mixture solutions with varying RI. Reprinted (**a**–**e**) with permission from Reference [110]. Reprinted (**f**,**g**) with permission from Reference [111].

The interference of the radiant and subradiant LSPR modes is usually realized by structural symmetry breaking and the optical field coupling among multiple metallic elements. In plasmonic sensors, Wu et al. studied an infrared asymmetric metamaterial based on Fano resonance [109]. They investigated the structural/spectroscopic and binding properties of monolayer protein by precisely tuning the structural resonance towards (away from) the target vibration resonance. The potential for multispectral biosensing was also demonstrated. Verellen et al. reported a combined nanocross and nanobarn structure as shown Figure 6a [110]. When the structure is illuminated at grazing incidence, the hybrid structure demonstrates an extra sharp resonance around 950 nm wavelength (Figure 6b), which is the dark quadrupole mode coupled weakly to dipolar radiation. The charge density plots in Figure 6c clearly illustrate the dipolar (red box) and quadrupolar (blue box) nature of the modes. In Figure 6d,e, RI sensitivities exceeding 1000 nm/RIU with a FOM reaching 5/RIU in the NIR are obtained.

The periodic metallic array structures such as nanoholes [112,113] and nanoslits [114,115] can also support Fano resonance. Shen et al. demonstrated a periodic array of gold mushrooms, and each mushroom was composed of a gold cap, photoresist pillar, and holy gold film as shown in Figure 6f [111]. In this study, the Fano resonance is caused by the interference between Wood’s anomaly of the periodic array and the LSPR of the individual gold unit, which results in a very high RI sensitivity of 1015 nm/RIU and a FOM up to 108 /RIU (Figure 6g). Although the FOM of this gold mushroom arrays regardless of prisms or gratings is comparable to the SPR sensors, the mode interference requires a certain incident angle or polarization of illuminated light.

Recently, the bound state in the continuum (BIC) has attracted much attention for their high-Q modes [116]. BIC was first proposed in 1929 by von Neumann and Wigner in electronic systems [117]. In the field of optics, BIC has been realized in waveguides [118] and photonic crystal structures [119,120]. Note that perfect BIC can not be excited, and the observed BIC usually collapses to Fano resonance category [121]. The BIC structure of high-Q modes can be used for optical lasers [122], photonic sensors [123,124,125] and nonlinear optics [126,127]. Due to the high damping loss, it is challenging to realize BIC in plasmonic structures. Liang et al. reported that anistropic plasmonic metasurfaces can support quasi-BIC collective resonant modes [128]. Azzam et al. realized BIC in a hybrid plasmonic-photonic structure leveraging a destructive interference of the photonic and the plasmonic modes. In this system, two types of BIC are realized including symmetry-protected BIC and off-Γ Friedrich-Wintgen BIC [129]. Recently, Meudt et al. demonstrated the hybrid photonic–plasmonic BIC system for sensors with a FOM as high as $1.43\times 10^{5}$/RIU [130]. Later, Shen et al. [131] reported a relative humidity (RH) sensors based on this hybrid mode platform, which exhibited wavelength sensitivity of 1.16 nm %RH${}^{-1}$ and a quick response time of a few seconds. Besides the ordered plasmonic nanostructures mentioned above, the disordered system without delicate design of structures is also promising for sensors with the advantages of low-cost and simple excitation schemes [132]. This system with rough surface can also increase the FOM through Fano-resonant transmission profile [133]. For RI sensing, the nanoporous gold platforms have shown high sensitivity of 15,679 nm/RIU [134].

### 3.4. 2D Materials-Integrated Metastructure for Optical Sensing

The plasmonic sensors mentioned above only consider components of novel metals, and in this Section 2D materials integrated with plasmonic metastructures will be introduced. Metallic nanoparticles (NPs) have some inherent disadvantages, such as high cost, poor stability and adsorption towards chemicals or biomolecules. The integration of 2D materials in plasmonic sensors is complementary to conventional metal structures due to their unusual electrical and optical properties [135]. 2D materials refer to layered materials with strong in-plane bonds and Van der Waals force between layers [136]. Since the first 2D atomic crystal graphene was discovered in 2004 [137], the research of graphene is developing rapidly in photonics and optoelectronics [138,139]. The rise of graphene materials have also stimulated the development of other 2D materials possessing intriguing properties, such as transition metal dichalcogenides (TMDCs) and black phosphorous (BP) [140,141].

Graphene is a single or a few layers of $sp^{2}$ carbon atoms arranged in a honeycomb lattice. For monolayer structure, graphene has no bandgap with an electronic band structure characterized by the linear cones; thus, the electrons behave as massless Dirac fermions. The surface conductivity of graphene is governed by Kubo formula [142]:

(12) $$\sigma \left(\omega ,\mu_{c},\tau ,T\right)=\sigma_{\mathrm{intra}}\left(\omega ,\mu_{c},\tau ,T\right)+\sigma_{\mathrm{inter}}\left(\omega ,\mu_{c},\tau ,T\right)$$

in which:

(13) $$\begin{array}{c} \sigma_{\mathrm{intra}}\left(\omega ,\mu_{c},\tau ,T\right)=\frac{je^{2}}{\pi \hbar^{2}\left(\omega -j2\Gamma \right)}\int_{0}^{\infty }\left(\frac{\partial f_{d}\left(\xi ,\mu_{c},T\right)}{\partial \xi }-\frac{\partial f_{d}\left(-\xi ,\mu_{c},T\right)}{\partial \xi }\right)\xi d\xi \\ \sigma_{\mathrm{inter}}\left(\omega ,\mu_{c},\tau ,T\right)=\frac{je^{2}\left(\omega -j2\Gamma \right)}{\pi \hbar^{2}}\int_{0}^{\infty }\frac{f_{d}\left(\xi ,\mu_{c},T\right)-f_{d}\left(-\xi ,\mu_{c},T\right)}{{\left(\omega -j2\Gamma \right)}^{2}-4{\left(\xi /\hbar \right)}^{2}}d\xi \end{array}$$

where $f_{d}\left(\xi ,\mu_{c},T\right)={\left(e^{\frac{\xi -\mu_{c}}{k_{B}T}}+1\right)}^{-1}$ is Fermi-Dirac distribution, ω is angular frequency, *e* is the electron charge, *T* is absolute temperature, ξ is electron dynamic energy, Γ is scattering rate and $\Gamma =2\tau^{-1}$, τ is the relaxation time, *ℏ* is the reduced Planck’s constant, $\mu_{c}$ is the graphene chemical potential, $k_{B}$ is Boltzmann constant. The first and second part in Equation (12) denote graphene intraband and interband transitions, respectively. When the imaginary part of graphene conductivity $\sigma_{i}$ in Equation (12) is positive, the graphene layer can be treated as a very thin metal film supporting transverse magnetic (TM) mode [143]. While for the case that the $\sigma_{i}$ is negative (the inter-band optical transition dominates), the weakly damped transverse electric (TE) SPR modes might appear [144]. Since the chemical potential of graphene is gauged by the electric gating and chemical doping, the optical response of graphene can be dynamically manipulated [145,146,147,148].

**Figure 7:**
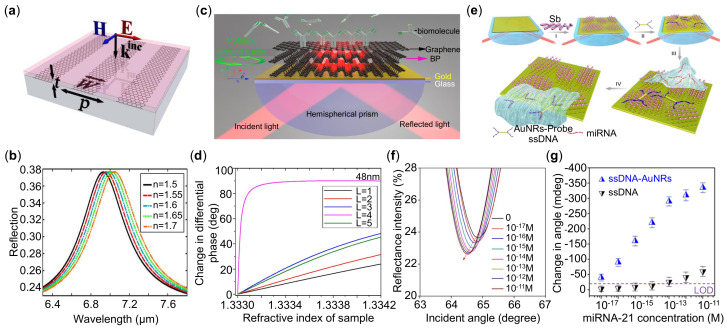
(**a**) Illustration of graphene ribbons. (**b**) Reflection spectra for graphene ribbons covered with different dielectric films at a constant thickness of 2 nm. (**c**) Schematic diagram of graphene–black phosphorous (BP) surface plasmon resonance (SPR) biosensor. (**d**) The change in differential phase with the variation of RI. (**e**) Fabrication process diagram of miRNA sensor integrated with antimonene nanomaterials. (**f**) Reflection spectra shift for different miRNA-21 concentrations assisted by AuNRs amplification. (**g**) SPR angle shift versus the miRNA-21 concentration. Reprinted (**a**,**b**) with permission from Reference [166]. Reprinted (**c**,**d**) with permission from Reference [168]. Reprinted (**e**–**g**) with permission from Reference [169].

In plasmonic sensors, graphene materials can support optical modes of both SPRs [149,150,151] and LSPRs [33,152,153,154]. Besides, graphene shows a higher adsorption efficiency to biomolecules via π-π interaction [33]. The first numerical work that combining graphene in SPR biosensor shows that the increasing numbers of graphene can enhance the sensitivity, which is resulted from the adsorbing of the biomolecules and optical response of graphene [155]. In addition, different photonic structures of graphene have been proposed for empowering the plasmonic sensing such as nanodisks [156], nanostripes [157,158] and other sub-wavelength structures [145]. Researchers also combine the graphene sheets with the metallic NPs [159,160,161,162] and dielectric/metallic gratings [163,164] for sensing applications. For example, Chen et al. [165] demonstrated a hybrid graphene-metal gratings for terhertz sensors with a FOM larger than 10. The periodic graphene nanoribbons as illustrated in Figure 7a have been extensively studied for mid- and far-IR plasmonic biosensing [166]. Compared with conventional metal plasmonic, the plasmon resonance of microstructured graphene can be tuned to the vibration frequency of target molecules [157,167]. Besides, the strong light confinement in graphene, which is two orders of magnitude higher than in metals, has boosted superior sensitivity in biomolecules detection as shown in Figure 7b [166].

Besides the extensively-studied graphene materials, BP consists of layered materials exhibiting strong in-plane anisotropy by their unique puckered structure [170]. This prominent anisotropic characteristic offers BP an extra degree of freedom in the design of optical devices [171]. Recently, a highly anisotropic and sensitive SPR biosensor composed of BP, graphene and gold thin film was proposed by Yuan et al. [168] as shown in Figure 7c. Phase-modulation approach is applied (differential phase between transverse-magnetic and transverse-electric polarized light) to characterize the performance of the plasmonic sensors. In the theoretical analysis, transfer matrix method and Fresnel equations are utilized to to study the phase and reflectivity changes. The absorption efficiency and energy loss in this architecture are balanced by optimizing the thickness of gold and BP film. In Figure 7d, it turns out that 4 layers of BP and 48 nm of gold coating will produce the sharpest phase variation with a sensitivity up to be $7.4914\times 10^{4}$ degree/RIU. Besides, due to the anisotropy of BP, this SPR biosensor exhibits tunable detection sensitivity by simply rotating this device. A relentless effort is being made to explore novel 2D materials for optical sensors. The recent developed antimonene exhibits strong spin–orbit coupling, tremendous stability and hydrophilicity. Bao et al. demonstrated an antimonene integrated SPR sensors as shown in Figure 7e [169]. The high sensitivity of this SPR sensor can be attributed to both the interaction of antimonene and single stranded DNA, and the coupling between the gold nanorods and propagating-SPR of the gold film. Specifically, the detection of miRNA-21 with a concentration as low as $10^{-17}$ M was observed as shown in Figure 7f. The detection limit of modified single-stranded DNA (ssDNA) (blue dots) is $10^{5}$ times lower than that using the non-modified ssDNA (black dots) in Figure 7g.

## 4. Optical Fiber-Based Plasmonic Sensors

Optical fiber technology developed rapidly in 1970s, which have stimulated the progresses of the modern fiber communications [172,173]. In addition to guiding the light wave in optical fibers, the propagating light can interact with outer surroundings, leading to the variation of optical signals; thus, optical fibers have also been used as sensing components [174]. Over the past years, optical fiber sensors have received much attention due to their advantages such as immune to electromagnetic interference, quick response, lightweight, and endurance in harsh environment, etc. [175]. To further enhance the fiber sensing performance, researchers have revolutionized the structural design and materials integration in optical fibers [138]. In this section, we will mainly focus on plasmonic fiber sensors, which should be dated backed to 1992 when Jorgenson et al. [176] first used fiber-optic SPR structures for chemical sensors. The plasmonic fiber sensors can not only improve the conventional fiber sensors’ performance by SPR and LSPR structures, but also allows for small sensing elements, simplified optical design and remote sensing [177]. The optical fiber-based plasmonic sensors are mainly categorized into three types, i.e., LSPR-fiber, SPR-fiber and 2D materials-fiber sensors, which will be discussed below.

### 4.1. Optical Fiber-LSPR RI Sensors

**Figure 8:**
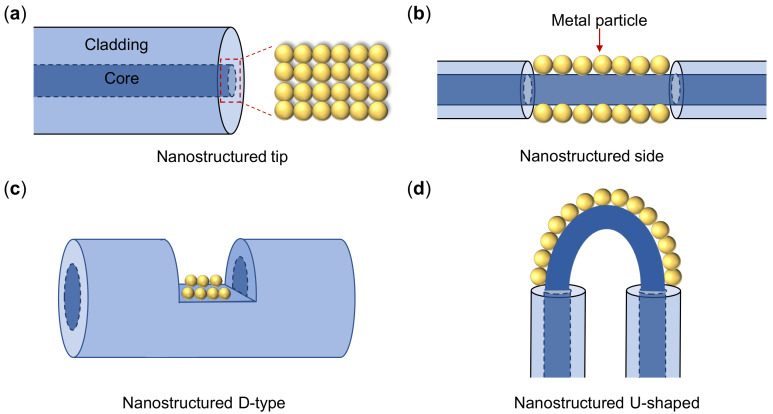
Schematic of typical structures used for fiber-LSPR sensors. (**a**) Plasmonic nanostructures on fiber tip, (**b**) Fiber side wall, (**c**) D-type fiber, (**d**) U-shaped fiber.

The representative configurations of optical fibers are high-refractive-index glass core surrounded by low-refractive-index cladding materials. This structure ensures that the propagating wave can be trapped inside the core of the fiber without leaking out. According to the supported mode numbers, the optical fiber can be divided into single mode fiber and multimode fiber (MMF), and usually the MMF has larger core radius. In order to couple the guiding wave into surface plasmons, the structure of fibers needs to be modified [177,178], as shown in Figure 8. Similar to the metastructure-based plasmonic sensors, the interrogation methods used in plasmonic fiber sensors including wavelength, intensity and phase measurements, in which the resonant wavelength monitoring is the most commonly employed method.

The excitations of LSPR in metal NPs are realized through the near-field coupling at the endface or side wall of optical fibers. The optical fiber tip is an attracting platform since it holds the advantages of free-space light couplings and remote light manipulations. The most common structures on the fiber tip includes metal NP arrays [179,180] and metal nanoholes (NHs) [181]. Jia et al. demonstrated the NHs array on the endface of a MMF exhibiting sensitivity of 559 nm/RIU [181]. In the propagating waveguide structure, the evanescent field leaks from the fiber core and is coupled with the NPs, which increases the sensitivity of the fiber sensor compared with that of a bare one. Cao et al. [182] immobilized two kinds of gold-NPs (nanospheres and nanorods) on the surface of a claddingless optical fiber. The sensitivity is 914 nm/RIU and 601 nm/RIU for nanospheres and nanorods, respectively. The fiber-LSPR sensors reported in this work are cost effective to produce; thus, they are potentially disposable after single use avoiding the cross-contaminations. For D-type fiber-LSPR sensors, Cennamo et al. [183] proposed five-branched gold nanostars in a plastic optical fiber, and the measured sensitivity was 580 nm/RIU. Song et al. [184] explored two shapes of NPs (triangular and sphere) deposited on U-shaped fibers, and they found that the performance of the triangular silver NPs sensor was higher than that of sphere structures with sensitivity of 1116.8 nm/RIU. We summarize the typical RI-sensing performance of different fiber-LSPR structures in Table 1, from which it is found that the U-shaped fiber-LSPR sensors show a higher sensitivity than that of other structured fibers.

### 4.2. Optical Fiber-SPR RI Sensors

Different from the fiber-LSPR, the excitation of SPR requires momentum matching; thus, the optical dispersion of fiber waveguide is of importance. The typical dispersion of SPR can be written as:

(14) $$k_{\mathrm{spp}}=\frac{\omega }{c}\sqrt{\frac{n_{a}^{2}\varepsilon_{m}}{n_{a}^{2}+\varepsilon_{m}}}$$

where $n_{a}$ denotes the refractive index of the analyte. The propagation of light in fiber is based on total internal reflection, and when the propagation constant is equal to $k_{\mathrm{SPP}}$, the SPR can be efficiently excited which is quite similar to the Kretschmann configurations. Compared with the prism-based SPR sensors, the optical fiber-SPR structures have the advantages of easier tuning and smaller bulk size. Note that the optical fibers have cylindrical shape and the optical modes possess spatially varying 3D-optical field; thus, only part of the energy inside the core can couple with SPR mode [177,195]. Overall, the design of fiber-SPR sensors includes mainly two steps: The first is modifying the shape of fiber such as removing the cladding, bending the fiber and grinding the fiber side or fiber tip to a certain angle; the second step is the metallic coating [177,196,197,198]. The former step supports the evanescent field escaping from the core of fiber to excite the SPR mode, the latter step greatly enhances the light-matter interaction.

**Figure 9:**
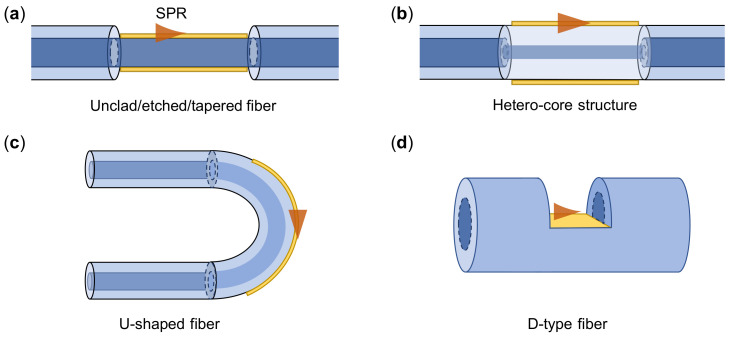
Schematics of different geometry-modified optical fiber-SPR sensors. (**a**) Unlad/etched/tapered fibers, (**b**) Hetero-core structure fibers, (**c**) U-shaped fibers, (**d**) D-type fibers.

Some types of geometry-modified optical fiber-SPR sensors are illustrated in Figure 9. In particular, Figure 9a displays the unclad/etched/tapered plasmonic fiber, and this hybrid claddingless fiber is the first reported for chemical sensor [176]. Tapered fibers can be produced by heating and stretching methods to draw from a standard optical fiber. Ding et al. [199] demonstrated SPR sensor based on a tapered coreless fiber with a high sensitivity of 2278.4 nm/RIU in the RI range of 1.33–1.391. The hetero-core fiber structure contains a single mode fiber between two MMFs as shown in Figure 9b. Due to the core mismatch, the core energy will leak out and interact with the surface metal film. Liu et al. [200] polished the traditional hetero-core structure as circular truncated cone shape and the SPR wavelength was varied with the polishing angle. They realized dual-channel sensing with sensitivity of 1980.77 nm/RIU and 4057.69 nm/RIU, respectively.

A more easier method to extract the light field inside the core is bending the fiber as shown in Figure 9c. Arcas et al. [201] presented a gold-coated U-shaped plastic fiber biosensor for *E. coli* bacteria detection. The intensity modulation in the U-shaped fiber by the gold SPR and bending loss were explored. For 70 nm and 100 nm gold-coated probes, the SPR effect was predominant and the bacteria concentrations as low as 1.5 ×$10^{3}$ colony-forming units (CFU)/mL was detected. The D-type fiber is conventionally fabricated by side-polishing technique to expose the fiber core and enhance the surface evanescent field. By depositing a metal film on the flat polished region, the surface guided SPR can be excited by a certain light polarization (Figure 9d). Dong et al. [202] proposed D-type few-mode fiber (FMF) based SPR sensors with a high sensitivity up to 4903 nm/RIU and a FOM of 46.1 /RIU. They claimed that when combined with mode-multiplexing technique, the FMF-SPR sensors could be used for high throughput biochemical analysis. Cao et al. [203] demonstrated a D-type low-index polymer fiber with SPR which had a record high sensitivity of 22,779 nm/RIU at 1.3350.

**Figure 10:**
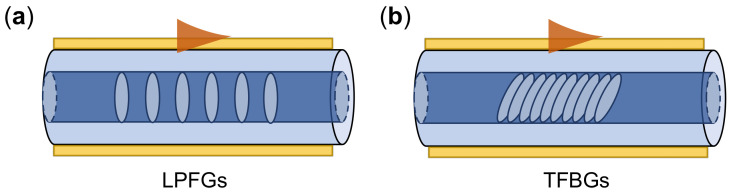
Schematics of the optical fiber grating-assisted SPR- fiber sensors. (**a**) Long period fiber gratings (LPFGs), (**b**) Tilted fiber Bragg gratings (TFBGs).

The fiber structures discussed above may suffer from the mechanical fragility due to the removed fiber claddings or reduced fiber diameters. The inline optical fiber grating (OFG) structures can overcome this problem. In the fiber gratings, the refractive index of fiber core along the propagation axis is periodically changed as shown in Figure 10. The conventional fabrication method of OFG is through UV laser interference patterns, and later other methods by phase mask inscription [204] and direct writing by femtosecond laser [205] are developed. The resonance wavelength of a uniform OFG is:

(15) $$\lambda_{m}=\left(n_{\mathrm{eff}}^{\mathrm{co}}-n_{\mathrm{eff}}^{\mathrm{cl}(m)}\right)\Lambda$$

where the $\lambda_{m}$ is the resonance wavelength of *m*-order cladding mode, $n_{\mathrm{eff}}^{\mathrm{co}}-n_{\mathrm{eff}}^{\mathrm{cl}(m)}$ denotes the effective difference between the core and *m*-order cladding mode, Λ is the period of the grating. Under the resonant condition, the lightwave is coupled from the core to the cladding, and excite the SPR mode efficiently.

To extract the light field from the fiber core to the cladding, the long period fiber gratings (LPFGs) can provide the matched reciprocal vector as shown in Figure 10a [206]. Hu et al. [207] numerically explored the LPFG coated with a thin metal film by the coupled-mode theory, and the calculated sensitivity is 1660 nm/RIU. The tilted fiber Bragg gratings (TFBGs) are another important fiber structures as displayed in Figure 10b. The remarkable characteristics of TFBGs distinguishing from the fiber Bragg gratings lies in the tilt grating angle, which brings multiple resonant couplings of cladding modes in addition to the Bragg reflection [208,209]. Guo et al. [210] demonstrated a highly sensitive detection of proteinuria using plasmonic TFBGs. They used a differential amplitude monitoring between the plasmonic and cut-off resonances, and they realized a protein detection sensitivity of 5.5 dB/(mg/ml) and a LOD of 1.5 × 10${}^{-3}$ mg/ml. Since the reflected Bragg mode can only be affected by temperature and strain effects which can serve as a self-reference spectra, the plasmonic-TFBG sensors show small temperature cross-sensitivity. Caucheteur et al. [211] used a plasmonic TFBG with a tilt angle near 37 degree to excite the SPR mode in the air cladding. This device could detect RI change of air as the pressure was varied with a sensitivity of 204 nm/RIU and RI resolution as low as $10^{-8}$/RIU. Baiad et al. [212] proposed concatenated plasmonic TFBG sensors with different tilt angles in a single optical fiber. The measured sensitivity is around 500 nm/RIU for the RI range from 1.40 to 1.44.

Besides the typical microstructured fiber aforementioned, some rationally designed fiber-SPR device, such as an eccentric-core fiber [213], HMM on D-type fiber [214], twin-core fiber [215], MMF/photonic crystal fiber/MMF [216] are proposed with impressive RI sensitivity in the range of 4000–9000 nm/RIU. Otherwise, the fabrication of these sensors becomes complicated with higher cost compared to traditional fiber-SPR sensors. The typical sensing performance of fiber-SPR devices are summarized in Table 2.

### 4.3. 2D Materials Integrated Plasmonic Fiber Sensors

Integrating 2D materials to fiber-optic plasmonic sensors is an effective strategy to increase the performance of sensors. On the one hand, 2D materials serve as protective layers of the novel metals accompanied with enhanced molecules adsorption. On the other, they can further increase the localized electromagnetic field. Graphene [228,229,230,231,232,233], TMDs [234,235] and BP [236] combined with different types of optical fibers sensors have been successfully demonstrated.

**Figure 11:**
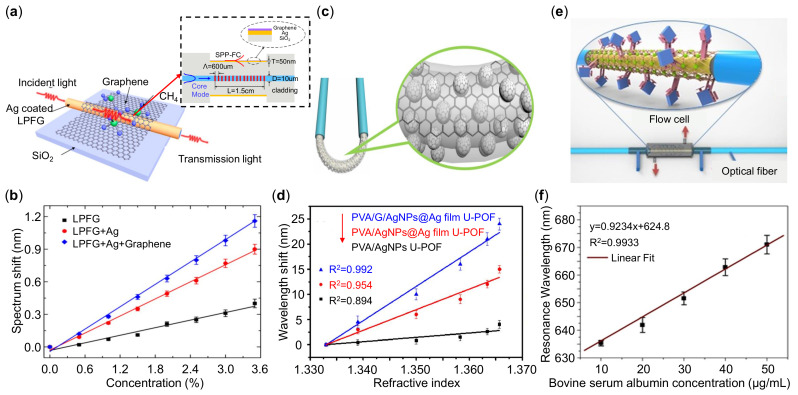
Plasmonic fiber sensors integrated with two-dimensional (2D) materials. (**a**) Schematic of the graphene-based LPFG-SPR sensor. Inset: longitudinal cross-section of the sensors. (**b**) Wavelength shift with the concentration of methane for LPFG (black), LPFG with Ag coating (red) and LPFG with Ag and graphene coating (blue) respectively. (**c**) Schematic diagram of U-shaped fiber coated with Ag NPs and graphene. (**d**) Wavelength shift as a function of RI for only Ag NPs (black), Ag NPs/Ag film (red) and Ag NPs/Ag film/graphene (blue) respectively. (**e**) Illustration of fiber coated with Au film and MoS${}_{2}$, and (**f**) wavelength shift with the concentration of bovine serum albumin solution. Reprinted (**a**,**b**) with permission from Reference [229]. Reprinted (**c**,**d**) with permission from Reference [194]. Reprinted (**e**,**f**) with permission from Reference [234].

Wei et al. [229] demonstrated a graphene-based LPFG-SPR sensor for high-sensitivity gas sensing as shown in Figure 11a. A metal film coated on the LPFG will enhance the surface optical field; however, it cannot adsorb molecules effectively. The graphene coating benefits for both the localized electromagnetic field and molecule adsorption efficiency. In Figure 11b, the graphene-based LPFG-SPR sensors have the best sensing performance, and the sensitivity is improved by 2.96 (1.31) times compared to a bare LPFG sensor (Ag-coated LPFG sensor). Jiang et al. [194] presented a U-shaped plastic optical fiber-SPR sensors based on graphene and silver hybrid structure as shown in Figure 11c. The U-shaped fiber deposited with graphene/Ag NPs/Ag film have an obviously higher sensitivity of 700.3 nm/RIU thanks to the biocompatibility and electric field enhancement of graphene. The TMD nanosheets hold great potential in biosensing applications for their large surface area and interaction with biomolecules [237]. Kaushik et al. [234] reported a MoS${}_{2}$-assisted biofunctionalized fiber-SPR biosensor as illustrated in Figure 11e. The antibodies were used for label-free detection of bovine serum albumin (BSA) protein. The fiber sensing performance with MoS${}_{2}$ is better than the one without MoS${}_{2}$. The synergic effects of MoS${}_{2}$ and gold film amplified the SPR signals with an improved detection limit of 0.29 μg/mL. Note that in most cases, 2D materials in plasmonic fiber sensors act as protecting layers accompanied with enhanced the adsorption efficiency and electromagnetic field. However, the improvement in sensing is not competitive and most of them are limited in the theoretical calculations.

## 5. Quantum Plasmonic Sensors

Although plasmonic sensors have been realized and commercialized successfully on different platforms, their sensitivity and associated precision are starting to reach the shot noise limit by quantum fluctuations of light [36,238]. To go beyond sensitivity limit by the SNL, the combination of quantum metrology with plasmonic sensing is increasingly being implemented in the field of chemistry and biology [62,239]. This emerging field is called “quantum plasmonic sensing” [19]. In this section, we briefly review the recent studies that have exploited quantum methods to improve the performance of plasmonic sensors.

**Figure 12:**
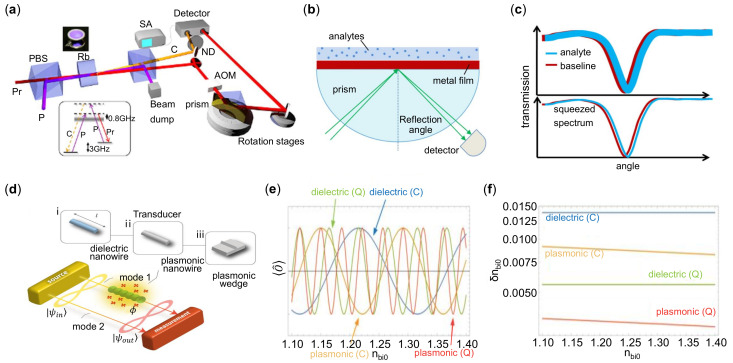
Quantum plasmonic intensity and phase sensors. (**a**) Experimental setup for sub-shot noise limit (SNL) plasmonic sensing. The inset shows the four-wave mixing scheme in Rb vapor. Two beams with different colors are used for probe (red) and reference (orange). (**b**) Schematic of Kretschmann SPR sensors with angular modulation. (**c**) Illustration of noise suppression via quantum-enhanced technology. (**d**) Schematic of two-mode interferometer with one arm in a nanowire waveguide. (**e**) Expectation values optimized for classical and quantum sensors. (**f**) Minimum resolution for classical and quantum sensors. Reprinted (**a**–**c**) with permission from Reference [241]. Reprinted (**d**–**f**) with permission from Reference [242].

Quantum resources have revolutionized the metrology in the field of quantum-enhanced sensors, in which the amplitude or phase squeezed light are widely used [240,241,242,243]. Pooser et al. [241] reported a heterodyne-based quantum plasmonic sensors as shown in Figure 12a. They realized a maximal quantum noise reduction of approximately 4.5 dB by combining the twin quantum correlated light fields with conventional Kretschmann geometry in Figure 12b. Note that the two-mode squeezed state was produced by pumping Rb vapor with degenerate four-wave mixing effects (inset of Figure 12a) [241,244]. Since the squeezed light can significantly reduce the shot noise, the sensing resolution is improved as illustrated in Figure 12c. Later, Lee et al. [245] studied two-mode beams with symmetric quantum statistical features combined with a SPR sensor, which notably enhanced the estimation precision of RI of the analytes. Zhao et al. [246] demonstrated an optical salinity quantum sensors in a tapered hetero-core plasmonic fiber. They realized a salinity sensitivity of 0.02204/‰with a ultra-resolution of 0.0016‰, exhibiting one order of magnitude higher than that of traditional optical fiber-SPR sensors.

The interferometric measurements are generally more sensitive than intensity-based detection under the same conditions. Thus, the quantum phase sensors are attracting attentions. Lee et al. [242] first studied a two-mode interferometer with quantum states input as shown in Figure 12d. In the interferometer, the sensing arm was composed of a silver nanowire waveguide as a plasmonic mode 1, and the reference mode 2 was on the other arm. The change of the biological medium surrounding the nanowire, will cause the relative phase change between the mode 1 and mode 2. In this work, both the classical and quantum sensing are taken into comparison: Classical sensing is modeled by a Mach-Zehnder interferometer with a coherent state input, and the NOON state input is considered in quantum sensing. The NOON state is an entangled quantum source which is often used in phase sensing to increase the precision [247,248], and it is defined as:

(16) $$|\Psi_{\mathrm{NOON}}\rangle =\frac{1}{\sqrt{2}}(|N,0\rangle +\left|0,N\rangle \right)$$

where *N* is the number of photons. When employing the NOON state in quantum phase sensing, it provides a RI-precision (Δn):

(17) $$\Delta n=\frac{1}{N}{\left|\frac{d\phi }{dn}\right|}^{-1}$$

where ϕ is the optical phase. While, the precision for classical measurement using a two-mode coherent state is:

(18) $$\Delta n=\frac{1}{\sqrt{N}}{\left|\frac{d\phi }{dn}\right|}^{-1}$$

Evidently, the NOON-state measurement improves the precision compared with coherent input by $\sqrt{N}$ from Equations (17) and (18) [242].

The nanowire in Figure 12d is investigated in respect of dielectric and metallic material (lossless) for comparison. As shown in Figure 12e, the expectation value for the quantum plasmonic sensor oscillates far more rapidly than that of the others, which means that a small change induces a large detectable variation of the measurement signal. In addition, the resolution is smallest for the quantum plasmonic sensing as shown in Figure 12f, which unambiguously exhibits the superiority of quantum sensing. Chen et al. [249] experimentally demonstrated the excitation and propagation of a NOON state (*N* = 2) in a silver nanowire, their results showed that the quantum sensing advantage was possible with only moderate loss.

Apart from the quantum metrology, the strong coupling effects between plasmonic nanostructures and quantum emitters can also be applied for plasmonic sensing [250]. The Hamiltonian of this system can be written as [239]:

(19) $$H=\left(\begin{array}{cc} \omega_{e}-i\gamma /2 & g\\ g & \omega_{p}-i\kappa /2 \end{array}\right)$$

where $\omega_{e}$ and $\omega_{p}$ are the angular frequency of the emitter and plasmon-polaritons respectively; γ and κ are their decay rates. The two eigenvalues are derived as

(20) $$\omega_{\pm }=\frac{\omega_{e}+\omega_{p\;}}{2}-\frac{i}{4}\left(\gamma +\kappa \right)\pm \sqrt{g^{2}+{\left(\frac{\Delta }{2}-\frac{i}{4}\left(\gamma_{-}\kappa \right)\right)}^{2}}$$

where $\Delta =\omega_{e}-\omega_{p}$ is the frequency detuning. The interaction of nanosturctures and quantum emitters can be classified in two types according to the coupling strength: weak coupling and strong coupling. For weak coupling ($|g|<\left|\gamma -\kappa \right|/4$), the coupling rate is smaller than the decay rate, and the Purcell effect dominates [251]. In the strong coupling regime ($|g|>\left|\gamma -\kappa \right|/4$), some quantum effects such as Rabi splitting [252,253,254], quantum entanglement [255] and optical Stark effect [256] can be observed. The strong coupling is achieved in various platforms, such as nanorod [257], bowtie [258], nanoparticle [259] and 2D atomic crystals system [260]. For the plasmonic sensing, various configurations such as quantum dot-metallic nanorod [261], quantum dot-metallic nanoshell [262] and quantum emitter/Ag-NR/silver nanowire composite [263] are proposed, which pave a way to quantum-enhanced single molecule detection.

**Figure 13:**
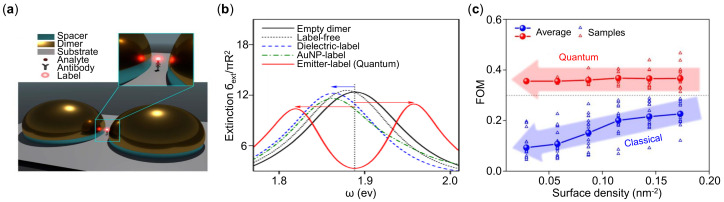
Quantum plasmonic sensors by strong coupling. (**a**) Schematic of the strong-coupling immunoassay setup. (**b**) Comparisons of performance for different plasmonic sensors. (**c**) FOM as a function of the surface density of analyte-emitter complexes. Reprinted (**a**–**c**) with permission from Reference [264].

Recently, Kongsuwan et al. [264] studied the strong coupling-based sensing that utilized the interactions between the quantum emitter label and plasmon-polaritons as shown Figure 13a. In this system, a hotspot is located between the nanoplasmonic dimer. The antigen on the antibody acts as analytes to be detected.

Conventional sensors with dielectric label and Au-NP label exhibit only shift of resonant spectra, while the quantum emitter label shows Rabi splitting, which is the signature of strong coupling as shown in Figure 13b. The sensitivity in strong coupling regime is defined as:

(21) $$S_{N}^{\mathrm{split}\;}=\frac{\delta \omega -|\Delta |}{N}$$

where $\delta \omega =\left|\omega_{+}-\omega_{-}\right|$, and *N* is the numbers of analytes. The proposed splitting-type sensing approach records an sensitivity enhancement of nearly 15-fold over the conventional shifting-type label-free plasmonic sensors. In the ensemble of analyte-complexes, they found that when the surface density was decreased, the FOM of classical regime dropped drastically while the quantum regime remained almost constant as shown in Figure 13c. This work shows great potential towards single-analyte detection. We note that for plasmonic sensors, the fundamental quantum properties of a plasmonic system, such as electron tunneling [238,265,266], nonlocal [267], and quantum surface response [268] are also exciting areas ready to be explored.

## 6. Conclusions and Outlook

In this work, we briefly reviewed the recent advances of surface plasmonic sensors. We mainly focused on the platform of planar metastructures and optical fibers, and discussed their sensing mechanisms, structural design and materials integration. Apart from the building blocks of only novel metals, the 2D materials integrated in plasmonic sensors is an emerging field which may revolutionize the way in sensing such as flexible and wearable biosensors [269]. From the classical measurement to quantum metrology, the emerging quantum plasmonics provide a new paradigm shift in optical sensing, which have realized unprecedented performance beyond the traditional methods. More realistic applications in biochemistry and medicine based on quantum technology are expected in the near future.

As for the future development of plasmonic sensors, we envision that there is still plenty of room to explore in the category of classical optics. Besides the platforms of planar metastructures and optical fibers, the whispering-gallery mode (WGM) microcavities and photonic crystals (PCs) have received much attention for their ultrahigh-Q factors and moderate mode volumes [43,270,271,272,273,274]. Integrating the plasmonic structures in WGM and PCs, the ultrahigh sensitivity for single-molecule and single-ion detection have been achieved [274,275,276]. In respect of the plasmonic materials, the most used noble metals such as silver and gold have some limitations for plasmonic devices considering their inherent optical loss, high cost (for gold) and oxidation issue (for silver). The emerging plasmonic materials such as aluminum [277,278,279] and sodium [280] may serve as possible alternatives for their advantages of low loss and relatively low cost. While the air instability is a serious issue for sodium-based plasmonic devices, researchers have demonstrated the stability of performance through appropriate packaging by quartz and epoxy [280]. In terms of fabrication method, electron beam lithography and focused ion beam are typical top-down methods to fabricate nanoscale plasmonic devices, which are not considered to be massive productive for their high cost and time consuming [281]. More cost-effective methods such as self-assembly (bottom-up) techniques [282,283] and nanoimprint lithography [277,284] hold great potentials for the commercialization. The assemblies of plasmonic NPs with controlled architectures have been demonstrated for quantitative surface enhanced Raman spectroscopy (SERS) [285]. For the optical interrogations, the conventional plasmonic sensors strongly rely on the spectral detection which requires sophisticated optical instruments. Tittl et al. [286] demonstrated an image-based molecular barcoding with a pixelated dielectric metasurface. The image-based sensing technique can resolve absorption fingerprints without the need for spectrometry, frequency scan, or mechanical components, which opens a new way for multi-analyte recognition with fast response [286,287,288,289]. For the analytes detection, besides the biological and chemical sensing, the operando monitoring electrochemical activity [290,291] is an attractive area for the development of green energy. We also note the importance of the emerging field of deep learning, which has shown great power in inversely designing the optical microstructures with enhanced electromagnetic response [292,293,294,295]. This advanced science and technology will endow more possibilities for the development of surface plasmonic sensors.

## Figures and Tables

**Table 1:** Summary of the experimental performances for fiber-LSPR RI sensors.

Fiber Structure	Plasmonic Structures	Interrogation	S nm/RIU	Reference
MMF tip	Au NHs	wavelength shift	487	[185]
fiber tip	Au NHs	wavelength shift	420 ± 83	[186]
fiber tip	Ag NPs	wavelength shift	387	[187]
claddingless MMF	Ag NPs	wavelength shift	914	[182]
claddingless MMF	Au nanorods	wavelength shift	771	[188]
tapered fiber	Ag NPs	intensity change	1.80 AU/RIU *	[189]
tapered fiber	Ag NPs	wavelength shift	51 nm/RIU	[190]
D-type fiber	Au NPs	wavelength shift	3074	[191]
D-type fiber	Au NPs	wavelength shift	580	[183]
U-shaped fiber	Ag NPs	wavelength shift	1198	[192]
U-shaped fiber	Ag NPs	wavelength shift	1116.8	[184]
U-shaped fiber	Au NPs + graphene	wavelength shift	1251.44	[193]
U-shaped fiber	Ag NPs + graphene	wavelength shift	700.3	[194]

* AU means absorbance unit.

**Table 2:** Performance of optical fiber-SPR sensors.

Fiber Structure	Physical Parameter	Sensitivity	FOM	Reference
claddingless fiber	protein concentration	2659.64 nm/RIU	∼21.2/RIU	[217]
tapered fiber	RI	1700 nm/RIU	∼13.9/RIU	[218]
tapered fiber	RI	2278.4 nm/RIU	∼12.6/RIU	[199]
S-tapered	RI	1441 nm/RIU	∼96.1/RIU	[219]
S-tapered fiber	RI	1882.4 nm/RIU	∼125.5/RIU	[220]
D-type fiber	RI	4284.8 nm/RIU	∼25.4/RIU	[221]
D-type fiber	RI	6328 nm/RIU	∼42.2/RIU	[202]
D-type fiber	RI	22,779 nm/RIU	61.2/RIU	[203]
D-type fiber	plumbum ions concentration	0.116 nm/ppm	$1.91\times 10^{-3}$/ppm	[222]
hetero-core fiber	RI	4057.69 nm/RIU	∼33.8/RIU	[200]
hetero-core fiber	RI	2933.25 nm /RIU/	530/RIU	[223]
U-shaped fiber	RI	220 nm/RIU	∼2.4/RIU	[224]
LPFG	strain	0.92 pm/με	-	[225]
multi-angle TFBG	RI	500 nm/RIU	-	[226]
TFBG	RI	536 nm/RIU	-	[227]

## Data Availability

Not applicable.

## References

[1] Wood R.W. (1902). On a remarkable case of uneven distribution of light in a diffraction grating spectrum. Philos. Mag. Ser..

[2] Mie G. (1908). Beiträge zur Optik trüber Medien, speziell kolloidaler Metallösungen. Ann. Phys..

[3] Fano U. (1941). The theory of anomalous diffraction gratings and of quasi-stationary waves on metallic surfaces (Sommerfeld’s waves). J. Opt. Soc. Am..

[4] Ritchie R.H. (1957). Plasma losses by fast electrons in thin films. Phys. Rev..

[5] Hessel A., Oliner A. (1965). A new theory of Wood’s anomalies on optical gratings. Appl. Opt..

[6] Fleischmann M., Hendra P.J., McQuillan A.J. (1974). Raman spectra of pyridine adsorbed at a silver electrode. Chem. Phys. Lett..

[7] Maier S.A., Brongersma M.L., Kik P.G., Meltzer S., Requicha A.A., Atwater H.A. (2001). Plasmonics—a route to nanoscale optical devices. Adv. Mater..

[8] Maier S.A. (2007). Plasmonics: Fundamentals and Applications.

[9] Xu Y., Gu C., Hou B., Lai Y., Li J., Chen H. (2013). Broadband asymmetric waveguiding of light without polarization limitations. Nat. Commun..

[10] Hao J., Wang J., Liu X., Padilla W.J., Zhou L., Qiu M. (2010). High performance optical absorber based on a plasmonic metamaterial. Appl. Phys. Lett..

[11] Oulton R.F., Sorger V.J., Zentgraf T., Ma R.M., Gladden C., Dai L., Bartal G., Zhang X. (2009). Plasmon lasers at deep subwavelength scale. Nature.

[12] Fang N., Lee H., Sun C., Zhang X. (2005). Sub–diffraction-limited optical imaging with a silver superlens. Science.

[13] Mayer K.M., Hafner J.H. (2011). Localized surface plasmon resonance sensors. Chem. Rev..

[14] Kretschmann E., Raether H. (1968). Radiative decay of non-radiative surface plasmons excited by light. Z. Naturforsch. A.

[15] Otto A. (1968). Excitation of nonradiative surface plasma waves in silver by the method of frustrated total reflection. Z. Phys. A Hadron. Nucl..

[16] Lin K., Lu Y., Chen J., Zheng R., Wang P., Ming H. (2008). Surface plasmon resonance hydrogen sensor based on metallic grating with high sensitivity. Opt. Express.

[17] Motogaito A., Mito S., Miyake H., Hiramatsu K. (2016). Detecting high-refractive-index media using surface plasmon sensor with one-dimensional metal diffraction grating. Opt. Photonics J..

[18] Dostalek J., Čtyroký J., Homola J., Brynda E., Skalský M., Nekvindova P., Špirková J., Škvor J., Schröfel J. (2001). Surface plasmon resonance biosensor based on integrated optical waveguide. Sens. Actuators B Chem..

[19] Lee C., Lawrie B., Pooser R., Lee K.G., Rockstuhl C., Tame M. (2021). Quantum plasmonic sensors. Chem. Rev..

[20] Barnes W.L., Dereux A., Ebbesen T.W. (2003). Surface plasmon subwavelength optics. Nature.

[21] Gramotnev D.K., Bozhevolnyi S.I. (2010). Plasmonics beyond the diffraction limit. Nat. Photonics.

[22] Šípová H., Homola J. (2013). Surface plasmon resonance sensing of nucleic acids: A review. Anal. Chim. Acta.

[23] Liang Y., Li L., Lu M., Yuan H., Long Z., Peng W., Xu T. (2018). Comparative investigation of sensing behaviors between gap and lattice plasmon modes in a metallic nanoring array. Nanoscale.

[24] Fan M., Andrade G.F., Brolo A.G. (2020). A review on recent advances in the applications of surface-enhanced Raman scattering in analytical chemistry. Anal. Chim. Acta.

[25] Rifat A.A., Ahmed R., Mahdiraji G.A., Adikan F.M. (2017). Highly sensitive D-shaped photonic crystal fiber-based plasmonic biosensor in visible to near-IR. IEEE Sens. J..

[26] Mauriz E., Calle A., Lechuga L.M., Quintana J., Montoya A., Manclus J. (2006). Real-time detection of chlorpyrifos at part per trillion levels in ground, surface and drinking water samples by a portable surface plasmon resonance immunosensor. Anal. Chim. Acta.

[27] Mauriz E., Dey P., Lechuga L.M. (2019). Advances in nanoplasmonic biosensors for clinical applications. Analyst.

[28] Hirsch L.R., Stafford R.J., Bankson J., Sershen S.R., Rivera B., Price R., Hazle J.D., Halas N.J., West J.L. (2003). Nanoshell-mediated near-infrared thermal therapy of tumors under magnetic resonance guidance. Proc. Natl. Acad. Sci. USA.

[29] Bülbül G., Hayat A., Andreescu S. (2015). Portable nanoparticle-based sensors for food safety assessment. Sensors.

[30] Homola J., Yee S.S., Gauglitz G. (1999). Surface plasmon resonance sensors. Sens. Actuators B Chem..

[31] Sreekanth K.V., Alapan Y., ElKabbash M., Ilker E., Hinczewski M., Gurkan U.A., De Luca A., Strangi G. (2016). Extreme sensitivity biosensing platform based on hyperbolic metamaterials. Nat. Mater..

[32] Sreekanth K.V., Alapan Y., ElKabbash M., Wen A.M., Ilker E., Hinczewski M., Gurkan U.A., Steinmetz N.F., Strangi G. (2016). Enhancing the angular sensitivity of plasmonic sensors using hyperbolic metamaterials. Adv. Opt. Mater..

[33] Ng S.P., Qiu G., Ding N., Lu X., Wu C.M.L. (2017). Label-free detection of 3-nitro-l-tyrosine with nickel-doped graphene localized surface plasmon resonance biosensor. Biosens. Bioelectron..

[34] Otto L.M., Mohr D.A., Johnson T.W., Oh S.H., Lindquist N.C. (2015). Polarization interferometry for real-time spectroscopic plasmonic sensing. Nanoscale.

[35] Yesilkoy F. (2019). Optical interrogation techniques for nanophotonic biochemical sensors. Sensors.

[36] Piliarik M., Homola J. (2009). Surface plasmon resonance (SPR) sensors: Approaching their limits?. Opt. Express.

[37] Wang X., Jefferson M., Hobbs P.C., Risk W.P., Feller B.E., Miller R.D., Knoesen A. (2011). Shot-noise limited detection for surface plasmon sensing. Opt. Express.

[38] Giovannetti V., Lloyd S., Maccone L. (2004). Quantum-enhanced measurements: Beating the standard quantum limit. Science.

[39] Giovannetti V., Lloyd S., Maccone L. (2011). Advances in quantum metrology. Nat. Photonics.

[40] Demkowicz-Dobrzański R., Jarzyna M., Kołodyński J. (2015). Quantum limits in optical interferometry. Prog. Optics..

[41] Degen C.L., Reinhard F., Cappellaro P. (2017). Quantum sensing. Rev. Mod. Phys..

[42] Braun D., Adesso G., Benatti F., Floreanini R., Marzolino U., Mitchell M.W., Pirandola S. (2018). Quantum-enhanced measurements without entanglement. Rev. Mod. Phys..

[43] Xu Y., Bai P., Zhou X., Akimov Y., Png C.E., Ang L.K., Knoll W., Wu L. (2019). Optical refractive index sensors with plasmonic and photonic structures: Promising and inconvenient truth. Adv. Opt. Mater..

[44] Gao M., Yang W., Wang Z., Lin S., Zhu J., Yang Z. (2020). Plasmonic resonance-linewidth shrinkage to boost biosensing. Photonics Res..

[45] Špačková B., Wrobel P., Bocková M., Homola J. (2016). Optical biosensors based on plasmonic nanostructures: A review. Proc. IEEE.

[46] Homola J., Piliarik M. (2006). Surface plasmon resonance (SPR) sensors. Surface Plasmon Resonance Based Sensors.

[47] Shrivastava A., Gupta V.B. (2011). Methods for the determination of limit of detection and limit of quantitation of the analytical methods. Chron. Young Sci..

[48] Jin M., Tang S.J., Chen J.H., Yu X.C., Shu H., Tao Y., Chen A.K., Gong Q., Wang X., Xiao Y.F. (2021). 1/f-noise-free optical sensing with an integrated heterodyne interferometer. Nat. Commun..

[49] Frascella G., Agne S., Khalili F.Y., Chekhova M.V. (2021). Overcoming detection loss and noise in squeezing-based optical sensing. NPJ Quantum Inform..

[50] Liu L., Zhang X., Kenney M., Su X., Xu N., Ouyang C., Shi Y., Han J., Zhang W., Zhang S. (2014). Broadband metasurfaces with simultaneous control of phase and amplitude. Adv. Mater..

[51] Wu P.C., Tsai W.Y., Chen W.T., Huang Y.W., Chen T.Y., Chen J.W., Liao C.Y., Chu C.H., Sun G., Tsai D.P. (2017). Versatile polarization generation with an aluminum plasmonic metasurface. Nano Lett..

[52] Yu N., Genevet P., Kats M.A., Aieta F., Tetienne J.P., Capasso F., Gaburro Z. (2011). Light propagation with phase discontinuities: Generalized laws of reflection and refraction. Science.

[53] Arbabi A., Arbabi E., Horie Y., Kamali S.M., Faraon A. (2017). Planar metasurface retroreflector. Nat. Photonics.

[54] Wang S., Wu P.C., Su V.C., Lai Y.C., Chu C.H., Chen J.W., Lu S.H., Chen J., Xu B., Kuan C.H. (2017). Broadband achromatic optical metasurface devices. Nat. Commun..

[55] Li L., Shuang Y., Ma Q., Li H., Zhao H., Wei M., Liu C., Hao C., Qiu C.W., Cui T.J. (2019). Intelligent metasurface imager and recognizer. Light Sci. Appl..

[56] Mueller J.B., Rubin N.A., Devlin R.C., Groever B., Capasso F. (2017). Metasurface polarization optics: Independent phase control of arbitrary orthogonal states of polarization. Phys. Rev. Lett..

[57] Yu N., Capasso F. (2014). Flat optics with designer metasurfaces. Nat. Mater..

[58] Wang Q., Zhang X., Xu Y., Tian Z., Gu J., Yue W., Zhang S., Han J., Zhang W. (2015). A broadband metasurface-based terahertz flat-lens array. Adv. Opt. Mater..

[59] Li H., Wang G., Hu G., Cai T., Qiu C., Xu H. (2020). 3D-printed curved metasurface with multifunctional wavefronts. Adv. Opt. Mater..

[60] La Spada L., Spooner C., Haq S., Hao Y. (2019). Curvilinear metasurfaces for surface wave manipulation. Sci. Rep..

[61] Zayats A.V., Smolyaninov I.I. (2003). Near-field photonics: Surface plasmon polaritons and localized surface plasmons. J. Opt. A-Pure Appl. Opt..

[62] Lee S., Song H., Ahn H., Kim S., Choi J.R., Kim K. (2021). Fiber-optic localized surface plasmon resonance sensors based on nanomaterials. Sensors.

[63] Khan A.U., Zhao S., Liu G. (2016). Key parameter controlling the sensitivity of plasmonic metal nanoparticles: Aspect ratio. J. Phys. Chem. C.

[64] Nugroho F.A.A., Albinsson D., Antosiewicz T.J., Langhammer C. (2020). Plasmonic metasurface for spatially resolved optical sensing in three dimensions. ACS Nano.

[65] Soehartono A.M., Tobing L.Y., Mueller A.D., Zhang D.H., Yong K. (2020). Hybrid transverse–longitudinal modes for high figure-of-merit localized plasmonic refractometric sensing in the visible spectrum. Adv. Opt. Mater..

[66] Jain P.K., El-Sayed M.A. (2010). Plasmonic coupling in noble metal nanostructures. Chem. Phys. Lett..

[67] Dykman L., Khlebtsov N. (2012). Gold nanoparticles in biomedical applications: Recent advances and perspectives. Chem. Soc. Rev..

[68] Estevez M.C., Otte M.A., Sepulveda B., Lechuga L.M. (2014). Trends and challenges of refractometric nanoplasmonic biosensors: A review. Anal. Chim. Acta.

[69] Khurana K., Jaggi N. (2021). Localized surface plasmonic properties of Au and Ag nanoparticles for sensors: A review. Plasmonics.

[70] Stewart M.E., Anderton C.R., Thompson L.B., Maria J., Gray S.K., Rogers J.A., Nuzzo R.G. (2008). Nanostructured plasmonic sensors. Chem. Rev..

[71] Chen Y., Ming H. (2012). Review of surface plasmon resonance and localized surface plasmon resonance sensor. Photonic Sens..

[72] Liu F., Song B., Su G., Liang O., Zhan P., Wang H., Wu W., Xie Y., Wang Z. (2018). Sculpting extreme electromagnetic field enhancement in free space for molecule sensing. Small.

[73] Cattoni A., Ghenuche P., Haghiri-Gosnet A.M., Decanini D., Chen J., Pelouard J.L., Collin S. (2011). *λ*^3^/1000 plasmonic nanocavities for biosensing fabricated by soft UV nanoimprint lithography. Nano Lett..

[74] Liu Z.Q., Shao H.B., Liu G.Q., Liu X.S., Zhou H.Q., Hu Y., Zhang X.N., Cai Z.J., Gu G. (2014). *λ*^3^/20000 plasmonic nanocavities with multispectral ultra-narrowband absorption for high-quality sensing. Appl. Phys. Lett..

[75] Bukasov R., Shumaker-Parry J.S. (2007). Highly tunable infrared extinction properties of gold nanocrescents. Nano Lett..

[76] Afkhami A., Kafrashi F., Ahmadi M., Madrakian T. (2015). A new chiral electrochemical sensor for the enantioselective recognition of naproxen enantiomers using l-cysteine self-assembled over gold nanoparticles on a gold electrode. RSC Adv..

[77] Droulias S., Bougas L. (2019). Surface plasmon platform for angle-resolved chiral sensing. ACS Photonics.

[78] Hentschel M., Schäferling M., Duan X., Giessen H., Liu N. (2017). Chiral plasmonics. Sci. Adv..

[79] Berova N., Polavarapu P.L., Nakanishi K., Woody R.W. (2011). Comprehensive Chiroptical Spectroscopy: Instrumentation, Methodologies, and Theoretical Simulations.

[80] Kakkanattu A., Eerqing N., Ghamari S., Vollmer F. (2021). Review of optical sensing and manipulation of chiral molecules and nanostructures with the focus on plasmonic enhancements. Opt. Express.

[81] Cao Z., Gao H., Qiu M., Jin W., Deng S., Wong K.Y., Lei D. (2020). Chirality transfer from sub-nanometer biochemical molecules to sub-micrometer plasmonic metastructures: Physiochemical mechanisms, biosensing, and bioimaging opportunities. Adv. Mater..

[82] Ben-Moshe A., Maoz B.M., Govorov A.O., Markovich G. (2013). Chirality and chiroptical effects in inorganic nanocrystal systems with plasmon and exciton resonances. Chem. Soc. Rev..

[83] Hendry E., Carpy T., Johnston J., Popland M., Mikhaylovskiy R., Lapthorn A., Kelly S., Barron L., Gadegaard N., Kadodwala M. (2010). Ultrasensitive detection and characterization of biomolecules using superchiral fields. Nat. Nanotechnol..

[84] García-Guirado J., Svedendahl M., Puigdollers J., Quidant R. (2018). Enantiomer-selective molecular sensing using racemic nanoplasmonic arrays. Nano Lett..

[85] Kelly C., Tullius R., Lapthorn A.J., Gadegaard N., Cooke G., Barron L.D., Karimullah A.S., Rotello V.M., Kadodwala M. (2018). Chiral plasmonic fields probe structural order of biointerfaces. J. Am. Chem. Soc..

[86] Lee H.E., Ahn H.Y., Mun J., Lee Y.Y., Kim M., Cho N.H., Chang K., Kim W.S., Rho J., Nam K.T. (2018). Amino-acid-and peptide-directed synthesis of chiral plasmonic gold nanoparticles. Nature.

[87] Tseng M.L., Lin Z., Kuo H.Y., Huang T., Huang Y., Chung T.L., Chu C.H., Huang J., Tsai D.P. (2019). Stress-induced 3D chiral fractal metasurface for enhanced and stabilized broadband near-field optical chirality. Adv. Opt. Mater..

[88] Zhao Y., Askarpour A.N., Sun L., Shi J., Li X., Alù A. (2017). Chirality detection of enantiomers using twisted optical metamaterials. Nat. Commun..

[89] Wu Z., Chen X., Wang M., Dong J., Zheng Y. (2018). High-performance ultrathin active chiral metamaterials. ACS Nano.

[90] Palermo G., Lio G.E., Esposito M., Ricciardi L., Manoccio M., Tasco V., Passaseo A., De Luca A., Strangi G. (2020). Biomolecular sensing at the interface between chiral metasurfaces and hyperbolic metamaterials. ACS Appl. Mater. Inter..

[91] Jeong H.H., Mark A.G., Alarcón-Correa M., Kim I., Oswald P., Lee T.C., Fischer P. (2016). Dispersion and shape engineered plasmonic nanosensors. Nat. Commun..

[92] Maoz B.M., Chaikin Y., Tesler A.B., Bar Elli O., Fan Z., Govorov A.O., Markovich G. (2013). Amplification of chiroptical activity of chiral biomolecules by surface plasmons. Nano Lett..

[93] Poddubny A., Iorsh I., Belov P., Kivshar Y. (2013). Hyperbolic metamaterials. Nat. Photonics.

[94] Liu Z., Lee H., Xiong Y., Sun C., Zhang X. (2007). Far-field optical hyperlens magnifying sub-diffraction-limited objects. Science.

[95] Poddubny A.N., Belov P.A., Kivshar Y.S. (2011). Spontaneous radiation of a finite-size dipole emitter in hyperbolic media. Phys. Rev. A.

[96] Ferrari L., Wu C., Lepage D., Zhang X., Liu Z. (2015). Hyperbolic metamaterials and their applications. Prog. Quant. Electron..

[97] Shekhar P., Atkinson J., Jacob Z. (2014). Hyperbolic metamaterials: Fundamentals and applications. Nano Converg..

[98] Kabashin A.V., Evans P., Pastkovsky S., Hendren W., Wurtz G.A., Atkinson R., Pollard R., Podolskiy V., Zayats A.V. (2009). Plasmonic nanorod metamaterials for biosensing. Nat. Mater..

[99] Sreekanth K.V., Krishna K.H., De Luca A., Strangi G. (2014). Large spontaneous emission rate enhancement in grating coupled hyperbolic metamaterials. Sci. Rep..

[100] Jiang L., Zeng S., Xu Z., Ouyang Q., Zhang D., Chong P.H.J., Coquet P., He S., Yong K. (2017). Multifunctional hyperbolic nanogroove metasurface for submolecular detection. Small.

[101] Fano U. (1961). Effects of configuration interaction on intensities and phase shifts. Phys. Rev..

[102] Luk’yanchuk B., Zheludev N.I., Maier S.A., Halas N.J., Nordlander P., Giessen H., Chong C.T. (2010). The Fano resonance in plasmonic nanostructures and metamaterials. Nat. Mater..

[103] Miroshnichenko A.E., Flach S., Kivshar Y.S. (2010). Fano resonances in nanoscale structures. Rev. Mod. Phys..

[104] Hao F., Sonnefraud Y., Dorpe P.V., Maier S.A., Halas N.J., Nordlander P. (2008). Symmetry breaking in plasmonic nanocavities: Subradiant LSPR sensing and a tunable Fano resonance. Nano Lett..

[105] Singh R., Al-Naib I.A., Koch M., Zhang W. (2011). Sharp Fano resonances in THz metamaterials. Opt. Express.

[106] Wu C., Khanikaev A.B., Shvets G. (2011). Broadband slow light metamaterial based on a double-continuum Fano resonance. Phys. Rev. Lett..

[107] Fan J.A., Wu C., Bao K., Bao J., Bardhan R., Halas N.J., Manoharan V.N., Nordlander P., Shvets G., Capasso F. (2010). Self-assembled plasmonic nanoparticle clusters. Science.

[108] Chen J., Gan F., Wang Y., Li G. (2018). Plasmonic sensing and modulation based on Fano resonances. Adv. Opt. Mater..

[109] Wu C., Khanikaev A.B., Adato R., Arju N., Yanik A.A., Altug H., Shvets G. (2012). Fano-resonant asymmetric metamaterials for ultrasensitive spectroscopy and identification of molecular monolayers. Nat. Mater..

[110] Verellen N., Van Dorpe P., Huang C., Lodewijks K., Vandenbosch G.A., Lagae L., Moshchalkov V.V. (2011). Plasmon line shaping using nanocrosses for high sensitivity localized surface plasmon resonance sensing. Nano Lett..

[111] Shen Y., Zhou J., Liu T., Tao Y., Jiang R., Liu M., Xiao G., Zhu J., Zhou Z.K., Wang X. (2013). Plasmonic gold mushroom arrays with refractive index sensing figures of merit approaching the theoretical limit. Nat. Commun..

[112] Zhan Y., Lei D.Y., Li X., Maier S.A. (2014). Plasmonic Fano resonances in nanohole quadrumers for ultra-sensitive refractive index sensing. Nanoscale.

[113] Yanik A.A., Cetin A.E., Huang M., Artar A., Mousavi S.H., Khanikaev A., Connor J.H., Shvets G., Altug H. (2011). Seeing protein monolayers with naked eye through plasmonic Fano resonances. Proc. Natl. Acad. Sci. USA.

[114] Zeng B., Gao Y., Bartoli F.J. (2014). Rapid and highly sensitive detection using Fano resonances in ultrathin plasmonic nanogratings. Appl. Phys. Lett..

[115] Lee K.L., Chen P.W., Wu S.H., Huang J.B., Yang S.Y., Wei P.K. (2012). Enhancing surface plasmon detection using template-stripped gold nanoslit arrays on plastic films. ACS Nano.

[116] Hsu C.W., Zhen B., Stone A.D., Joannopoulos J.D., Soljačić M. (2016). Bound states in the continuum. Nat. Rev. Mater..

[117] Von Neumann J., Wigner E. (1929). No crossing rule. Z. Phys..

[118] Plotnik Y., Peleg O., Dreisow F., Heinrich M., Nolte S., Szameit A., Segev M. (2011). Experimental observation of optical bound states in the continuum. Phys. Rev. Lett..

[119] Hsu C.W., Zhen B., Lee J., Chua S.L., Johnson S.G., Joannopoulos J.D., Soljačić M. (2013). Observation of trapped light within the radiation continuum. Nature.

[120] Jin J., Yin X., Ni L., Soljačić M., Zhen B., Peng C. (2019). Topologically enabled ultrahigh-Q guided resonances robust to out-of-plane scattering. Nature.

[121] Azzam S.I., Kildishev A.V. (2021). Photonic bound states in the continuum: From basics to applications. Adv. Opt. Mater..

[122] Kodigala A., Lepetit T., Gu Q., Bahari B., Fainman Y., Kanté B. (2017). Lasing action from photonic bound states in continuum. Nature.

[123] Leitis A., Tittl A., Liu M., Lee B.H., Gu M.B., Kivshar Y.S., Altug H. (2019). Angle-multiplexed all-dielectric metasurfaces for broadband molecular fingerprint retrieval. Sci. Adv..

[124] Srivastava Y.K., Ako R.T., Gupta M., Bhaskaran M., Sriram S., Singh R. (2019). Terahertz sensing of 7 nm dielectric film with bound states in the continuum metasurfaces. Appl. Phys. Lett..

[125] Romano S., Zito G., Torino S., Calafiore G., Penzo E., Coppola G., Cabrini S., Rendina I., Mocella V. (2018). Label-free sensing of ultralow-weight molecules with all-dielectric metasurfaces supporting bound states in the continuum. Photonics Res..

[126] Liu Z., Xu Y., Lin Y., Xiang J., Feng T., Cao Q., Li J., Lan S., Liu J. (2019). High-Q quasibound states in the continuum for nonlinear metasurfaces. Phys. Rev. Lett..

[127] Koshelev K., Kruk S., Melik-Gaykazyan E., Choi J.H., Bogdanov A., Park H.G., Kivshar Y. (2020). Subwavelength dielectric resonators for nonlinear nanophotonics. Science.

[128] Liang Y., Koshelev K., Zhang F., Lin H., Lin S., Wu J., Jia B., Kivshar Y. (2020). Bound states in the continuum in anisotropic plasmonic metasurfaces. Nano Lett..

[129] Azzam S.I., Shalaev V.M., Boltasseva A., Kildishev A.V. (2018). Formation of bound states in the continuum in hybrid plasmonic-photonic systems. Phys. Rev. Lett..

[130] Meudt M., Bogiadzi C., Wrobel K., Görrn P. (2020). Hybrid photonic–plasmonic bound states in continuum for enhanced light manipulation. Adv. Opt. Mater..

[131] Shen Y., Wang G., Zou Q., She X., Cai D., Jin C. (2021). Ultrasensitive dual-mode humidity detection using a plasmonic-photonic hybrid waveguide. Adv. Opt. Mater..

[132] Koya A.N., Zhu X., Ohannesian N., Yanik A.A., Alabastri A., Proietti Zaccaria R., Krahne R., Shih W.C., Garoli D. (2021). Nanoporous metals: From plasmonic properties to applications in enhanced spectroscopy and photocatalysis. ACS Nano.

[133] Zhu X., Cao N., Thibeault B.J., Pinsky B., Yanik A.A. (2020). Mechanisms of Fano-resonant biosensing: Mechanical loading of plasmonic oscillators. Opt. Commun..

[134] Garoli D., Calandrini E., Giovannini G., Hubarevich A., Caligiuri V., De Angelis F. (2019). Nanoporous gold metamaterials for high sensitivity plasmonic sensing. Nanoscale Horiz..

[135] Zhou J., Yang T., Chen J., Wang C., Zhang H., Shao Y. (2020). Two-dimensional nanomaterial-based plasmonic sensing applications: Advances and challenges. Coordin. Chem. Rev..

[136] Novoselov K.S., Jiang D., Schedin F., Booth T., Khotkevich V., Morozov S., Geim A.K. (2005). Two-dimensional atomic crystals. Proc. Natl. Acad. Sci. USA.

[137] Novoselov K.S., Geim A.K., Morozov S.V., Jiang D., Zhang Y., Dubonos S.V., Grigorieva I.V., Firsov A.A. (2004). Electric field effect in atomically thin carbon films. Science.

[138] Chen J.H., Xiong Y.F., Xu F., Lu Y.Q. (2021). Silica optical fiber integrated with two-dimensional materials: Towards opto-electro-mechanical technology. Light Sci. Appl..

[139] Tan T., Jiang X., Wang C., Yao B., Zhang H. (2020). 2D material optoelectronics for information functional device applications: Status and challenges. Adv. Sci..

[140] Bhimanapati G.R., Lin Z., Meunier V., Jung Y., Cha J., Das S., Xiao D., Son Y., Strano M.S., Cooper V.R. (2015). Recent advances in two-dimensional materials beyond graphene. ACS Nano.

[141] Butler S.Z., Hollen S.M., Cao L., Cui Y., Gupta J.A., Gutiérrez H.R., Heinz T.F., Hong S.S., Huang J., Ismach A.F. (2013). Progress, challenges, and opportunities in two-dimensional materials beyond graphene. ACS Nano.

[142] Hanson G.W. (2008). Dyadic Green’s functions and guided surface waves for a surface conductivity model of graphene. J. Appl. Phys..

[143] He X.Y., Tao J., Meng B. (2013). Analysis of graphene TE surface plasmons in the terahertz regime. Nanotechnology.

[144] Mikhailov S.A., Ziegler K. (2007). New electromagnetic mode in graphene. Phys. Rev. Lett..

[145] Islam M., Sultana J., Biabanifard M., Vafapour Z., Nine M., Dinovitser A., Cordeiro C., Ng B.H., Abbott D. (2020). Tunable localized surface plasmon graphene metasurface for multiband superabsorption and terahertz sensing. Carbon.

[146] Mak K.F., Sfeir M.Y., Wu Y., Lui C.H., Misewich J.A., Heinz T.F. (2008). Measurement of the optical conductivity of graphene. Phys. Rev. Lett..

[147] Novoselov K.S., Geim A. (2007). The rise of graphene. Nat. Mater..

[148] Liu H., Liu Y., Zhu D. (2011). Chemical doping of graphene. J. Mater. Chem..

[149] Chen J., Badioli M., Alonso-González P., Thongrattanasiri S., Huth F., Osmond J., Spasenović M., Centeno A., Pesquera A., Godignon P. (2012). Optical nano-imaging of gate-tunable graphene plasmons. Nature.

[150] Ni G., McLeod D.A., Sun Z., Wang L., Xiong L., Post K., Sunku S., Jiang B.Y., Hone J., Dean C.R. (2018). Fundamental limits to graphene plasmonics. Nature.

[151] Fan Y., Shen N., Zhang F., Zhao Q., Wu H., Fu Q., Wei Z., Li H., Soukoulis C.M. (2019). Graphene plasmonics: A platform for 2D optics. Adv. Opt. Mater..

[152] Velichko E.A. (2016). Evaluation of a graphene-covered dielectric microtube as a refractive-index sensor in the terahertz range. J. Opt..

[153] Hu F., Luan Y., Fei Z., Palubski I., Goldflam M., Dai S., Wu J.S., Post K., Janssen G., Fogler M. (2017). Imaging the localized plasmon resonance modes in graphene nanoribbons. Nano Lett..

[154] Wang W., Xiao S., Mortensen N.A. (2016). Localized plasmons in bilayer graphene nanodisks. Phys. Rev. B.

[155] Wu L., Chu H., Koh W., Li E. (2010). Highly sensitive graphene biosensors based on surface plasmon resonance. Opt. Express.

[156] Cen C., Lin H., Huang J., Liang C., Chen X., Tang Y., Yi Z., Ye X., Liu J., Yi Y. (2018). A tunable plasmonic refractive index sensor with nanoring-strip graphene arrays. Sensors.

[157] Rodrigo D., Limaj O., Janner D., Etezadi D., De Abajo F.J.G., Pruneri V., Altug H. (2015). Mid-infrared plasmonic biosensing with graphene. Science.

[158] Hu H., Yang X., Zhai F., Hu D., Liu R., Liu K., Sun Z., Dai Q. (2016). Far-field nanoscale infrared spectroscopy of vibrational fingerprints of molecules with graphene plasmons. Nat. Commun..

[159] Reed J.C., Zhu H., Zhu A.Y., Li C., Cubukcu E. (2012). Graphene-enabled silver nanoantenna sensors. Nano Lett..

[160] Wang P., Liang O., Zhang W., Schroeder T., Xie Y. (2013). Ultra-sensitive graphene-plasmonic hybrid platform for label-free detection. Adv. Mater..

[161] Cittadini M., Bersani M., Perrozzi F., Ottaviano L., Wlodarski W., Martucci A. (2014). Graphene oxide coupled with gold nanoparticles for localized surface plasmon resonance based gas sensor. Carbon.

[162] Hong Q., Luo J., Wen C., Zhang J., Zhu Z., Qin S., Yuan X. (2019). Hybrid metal-graphene plasmonic sensor for multi-spectral sensing in both near-and mid-infrared ranges. Opt. Express.

[163] Gao W., Shu J., Qiu C., Xu Q. (2012). Excitation of plasmonic waves in graphene by guided-mode resonances. ACS Nano.

[164] Wu T., Luo Y., Wei L. (2017). Mid-infrared sensing of molecular vibrational modes with tunable graphene plasmons. Opt. Lett..

[165] Chen Z.x., Chen J.H., Wu Z.J., Hu W., Zhang X.J., Lu Y.Q. (2014). Tunable Fano resonance in hybrid graphene-metal gratings. Appl. Phys. Lett..

[166] Vasić B., Isić G., Gajić R. (2013). Localized surface plasmon resonances in graphene ribbon arrays for sensing of dielectric environment at infrared frequencies. J. Appl. Phys..

[167] Kim J., Son H., Cho D.J., Geng B., Regan W., Shi S., Kim K., Zettl A., Shen Y.R., Wang F. (2012). Electrical control of optical plasmon resonance with graphene. Nano Lett..

[168] Yuan Y., Yu X., Ouyang Q., Shao Y., Song J., Qu J., Yong K.T. (2018). Highly anisotropic black phosphorous-graphene hybrid architecture for ultrassensitive plasmonic biosensing: Theoretical insight. 2D Mater..

[169] Xue T., Liang W., Li Y., Sun Y., Xiang Y., Zhang Y., Dai Z., Duo Y., Wu L., Qi K. (2019). Ultrasensitive detection of miRNA with an antimonene-based surface plasmon resonance sensor. Nat. Commun..

[170] Xia F., Wang H., Jia Y. (2014). Rediscovering black phosphorus as an anisotropic layered material for optoelectronics and electronics. Nat. Commun..

[171] Liu Z., Aydin K. (2016). Localized surface plasmons in nanostructured monolayer black phosphorus. Nano Lett..

[172] Kao K.C., Hockham G.A. (1986). Dielectric-fibre surface waveguides for optical frequencies. IEE Proc. J.

[173] Keiser G. (2003). Optical fiber communications. Wiley Encyclopedia of Telecommunications.

[174] Culshaw B. (2004). Optical fiber sensor technologies: Opportunities and-perhaps-pitfalls. J. Lightw. Technol..

[175] Chen J.H., Li D.R., Xu F. (2019). Optical microfiber sensors: Sensing mechanisms, and recent advances. J. Lightw. Technol..

[176] Jorgenson R.C., Yee S.S. (1993). A fiber-optic chemical sensor based on surface plasmon resonance. Sens. Actuators B Chem..

[177] Caucheteur C., Guo T., Albert J. (2015). Review of plasmonic fiber optic biochemical sensors: Improving the limit of detection. Anal. Bioanal. Chem..

[178] Urrutia A., Goicoechea J., Arregui F.J. (2015). Optical fiber sensors based on nanoparticle-embedded coatings. J. Sens..

[179] Jeong H.H., Erdene N., Park J.H., Jeong D.H., Lee H.Y., Lee S.K. (2013). Real-time label-free immunoassay of interferon-gamma and prostate-specific antigen using a fiber-optic localized surface plasmon resonance sensor. Biosens. Bioelectron..

[180] Kaye S., Zeng Z., Sanders M., Chittur K., Koelle P.M., Lindquist R., Manne U., Lin Y., Wei J. (2017). Label-free detection of DNA hybridization with a compact LSPR-based fiber-optic sensor. Analyst.

[181] Jia P., Yang J. (2013). Integration of large-area metallic nanohole arrays with multimode optical fibers for surface plasmon resonance sensing. Appl. Phys. Lett..

[182] Cao J., Tu M.H., Sun T., Grattan K.T. (2013). Wavelength-based localized surface plasmon resonance optical fiber biosensor. Sens. Actuators B Chem..

[183] Cennamo N., D’Agostino G., Donà A., Dacarro G., Pallavicini P., Pesavento M., Zeni L. (2013). Localized surface plasmon resonance with five-branched gold nanostars in a plastic optical fiber for bio-chemical sensor implementation. Sensors.

[184] Song H., Zhang H., Sun Z., Ren Z., Yang X., Wang Q. (2019). Triangular silver nanoparticle U-bent fiber sensor based on localized surface plasmon resonance. AIP Adv..

[185] Zhao E., Jia P., Ebendorff-Heidepriem H., Li H., Huang P., Liu D., Yang X., Liu L., Guan C. (2017). Localized surface plasmon resonance sensing structure based on gold nanohole array on beveled fiber edge. Nanotechnology.

[186] Polley N., Basak S., Hass R., Pacholski C. (2019). Fiber optic plasmonic sensors: Providing sensitive biosensor platforms with minimal lab equipment. Biosens. Bioelectron..

[187] Chen J., Shi S., Su R., Qi W., Huang R., Wang M., Wang L., He Z. (2015). Optimization and application of reflective LSPR optical fiber biosensors based on silver nanoparticles. Sensors.

[188] Cao J., Galbraith E.K., Sun T., Grattan K.T. (2012). Cross-comparison of surface plasmon resonance-based optical fiber sensors with different coating structures. IEEE Sens. J..

[189] Semwal V., Gupta B.D. (2019). Experimental studies on the sensitivity of the propagating and localized surface plasmon resonance-based tapered fiber optic refractive index sensors. Appl. Opt..

[190] Lin H.Y., Huang C.H., Cheng G.L., Chen N.K., Chui H.C. (2012). Tapered optical fiber sensor based on localized surface plasmon resonance. Opt. Express.

[191] Niu L.Y., Wang Q., Jing J.Y., Zhao W.M. (2019). Sensitivity enhanced D-type large-core fiber SPR sensor based on Gold nanoparticle/Au film co-modification. Opt. Commun..

[192] Zhang C., Li Z., Jiang S.Z., Li C.H., Xu S.C., Yu J., Li Z., Wang M.H., Liu A.H., Man B.Y. (2017). U-bent fiber optic SPR sensor based on graphene/AgNPs. Sens. Actuators B Chem..

[193] Li C., Li Z., Li S., Zhang Y., Sun B., Yu Y., Ren H., Jiang S., Yue W. (2020). LSPR optical fiber biosensor based on a 3D composite structure of gold nanoparticles and multilayer graphene films. Opt. Express.

[194] Jiang S., Li Z., Zhang C., Gao S., Li Z., Qiu H., Li C., Yang C., Liu M., Liu Y. (2017). A novel U-bent plastic optical fibre local surface plasmon resonance sensor based on a graphene and silver nanoparticle hybrid structure. J. Phys. D Appl. Phys..

[195] Sharma A.K., Jha R., Gupta B. (2007). Fiber-optic sensors based on surface plasmon resonance: A comprehensive review. IEEE Sens. J..

[196] Klantsataya E., Jia P., Ebendorff-Heidepriem H., Monro T.M., François A. (2017). Plasmonic fiber optic refractometric sensors: From conventional architectures to recent design trends. Sensors.

[197] Lee B., Roh S., Park J. (2009). Current status of micro-and nano-structured optical fiber sensors. Opt. Fiber Technol..

[198] Sharma A.K., Marques C. (2019). Design and performance perspectives on fiber optic sensors with plasmonic nanostructures and gratings: A review. IEEE Sens. J.

[199] Ding Z.W., Lang T.T., Wang Y., Zhao C.L. (2017). Surface plasmon resonance refractive index sensor based on tapered coreless optical fiber structure. J. Lightw. Technol..

[200] Liu Z., Liu L., Zhu Z., Zhang Y., Wei Y., Zhang Y., Yang J., Yuan L. (2017). Dual-channel surface plasmon resonance refractive index sensor based on modified hetero-core structure fiber. Opt. Commun..

[201] Arcas A.D.S., Dutra F.D.S., Allil R.C., Werneck M.M. (2018). Surface plasmon resonance and bending loss-based U-shaped plastic optical fiber biosensors. Sensors.

[202] Dong J., Zhang Y., Wang Y., Yang F., Hu S., Chen Y., Zhu W., Qiu W., Guan H., Lu H. (2019). Side-polished few-mode fiber based surface plasmon resonance biosensor. Opt. Express.

[203] Cao S., Shao Y., Wang Y., Wu T., Zhang L., Huang Y., Zhang F., Liao C., He J., Wang Y. (2018). Highly sensitive surface plasmon resonance biosensor based on a low-index polymer optical fiber. Opt. Express.

[204] Becker M., Elsmann T., Latka I., Rothhardt M., Bartelt H. (2015). Chirped phase mask interferometer for fiber Bragg grating array inscription. J. Lightw. Technol..

[205] Martinez A., Dubov M., Khrushchev I., Bennion I. (2004). Direct writing of fibre Bragg gratings by femtosecond laser. Electron. Lett..

[206] Rego G. (2013). A review of refractometric sensors based on long period fibre gratings. Sci. World J..

[207] Hu H.F., Deng Z.Q., Zhao Y., Li J., Wang Q. (2014). Sensing properties of long period fiber grating coated by silver film. IEEE Photonic. Tech. L..

[208] Erdogan T., Sipe J. (1996). Tilted fiber phase gratings. JOSA A.

[209] Guo T. (2017). Fiber grating-assisted surface plasmon resonance for biochemical and electrochemical sensing. J. Lightw. Technol..

[210] Guo T., Liu F., Liang X., Qiu X., Huang Y., Xie C., Xu P., Mao W., Guan B.O., Albert J. (2016). Highly sensitive detection of urinary protein variations using tilted fiber grating sensors with plasmonic nanocoatings. Biosens. Bioelectron..

[211] Caucheteur C., Guo T., Liu F., Guan B.O., Albert J. (2016). Ultrasensitive plasmonic sensing in air using optical fibre spectral combs. Nat. Commun..

[212] Baiad M.D., Kashyap R. (2015). Concatenation of surface plasmon resonance sensors in a single optical fiber using tilted fiber Bragg gratings. Opt. Lett..

[213] Liu Z., Wei Y., Zhang Y., Liu C., Zhang Y., Zhao E., Yang J., Liu C., Yuan L. (2015). Distributed fiber surface plasmon resonance sensor based on the incident angle adjusting method. Opt. Lett..

[214] Hu S., Chen Y., Chen Y., Chen L., Zheng H., Azeman N.H., Liu M.X., Liu G.S., Luo Y., Chen Z. (2020). High-performance fiber plasmonic sensor by engineering the dispersion of hyperbolic metamaterials composed of Ag/TiO_2_. Opt. Express.

[215] Liu Z., Wei Y., Zhang Y., Zhang Y., Zhao E., Yang J., Yuan L. (2015). Twin-core fiber SPR sensor. Opt. Lett..

[216] Wang Y., Huang Q., Zhu W., Yang M., Lewis E. (2018). Novel optical fiber SPR temperature sensor based on MMF-PCF-MMF structure and gold-PDMS film. Opt. Express.

[217] Wang W., Mai Z., Chen Y., Wang J., Li L., Su Q., Li X., Hong X. (2017). A label-free fiber optic SPR biosensor for specific detection of C-reactive protein. Sci. Rep..

[218] Cennamo N., Arcadio F., Minardo A., Montemurro D., Zeni L. (2020). Experimental characterization of plasmonic sensors based on lab-built tapered plastic optical fibers. Appl. Sci..

[219] Wang G., Li Z., Wang J., Shen J., Zhang M., Huang M. (2020). Fabrication and sensing characterization of an S-tapered fiber probe. AIP Adv..

[220] Chen C., Yang R., Zhang X.Y., Wei W.H., Guo Q., Zhang X., Qin L., Ning Y.Q., Yu Y.S. (2018). Compact refractive index sensor based on an S-tapered fiber probe. Opt. Mater. Express.

[221] Liu L., Deng S., Zheng J., Yuan L., Deng H., Teng C. (2021). An enhanced plastic optical fiber-based surface plasmon resonance sensor with a double-sided polished structure. Sensors.

[222] Alwahib A.A., Alhasan S.F., Yaacob M.H., Lim H.N., Mahdi M.A. (2020). Surface plasmon resonance sensor based on D-shaped optical fiber using fiberbench rotating wave plate for sensing pb ions. Optik.

[223] Zhang R., Pu S., Li X. (2019). Gold-film-thickness dependent SPR refractive index and temperature sensing with hetero-core optical fiber structure. Sensors.

[224] Paliwal N., Punjabi N., John J., Mukherji S. (2016). Design and fabrication of lossy mode resonance based U-shaped fiber optic refractometer utilizing dual sensing phenomenon. J. Lightw. Technol..

[225] Gu Z., Lan J., Gao K. (2016). Characteristics of plasmon coupling mode in SPR based LPFG. Opt. Quant. Electron..

[226] Chen X., Xu J., Zhang X., Guo T., Guan B.O. (2017). Wide range refractive index measurement using a multi-angle tilted fiber Bragg grating. IEEE Photonic. Tech. L..

[227] Liu F., Zhang X., Li K., Guo T., Ianoul A., Albert J. (2021). Discrimination of bulk and surface refractive index change in plasmonic sensors with narrow bandwidth resonance combs. ACS Sensors.

[228] Wang Q., Wang B.T. (2018). Surface plasmon resonance biosensor based on graphene oxide/silver coated polymer cladding silica fiber. Sens. Actuators B Chem..

[229] Wei W., Nong J., Zhang G., Tang L., Jiang X., Chen N., Luo S., Lan G., Zhu Y. (2017). Graphene-based long-period fiber grating surface plasmon resonance sensor for high-sensitivity gas sensing. Sensors.

[230] Zhang N.M.Y., Li K., Shum P.P., Yu X., Zeng S., Wu Z., Wang Q.J., Yong K.T., Wei L. (2017). Hybrid graphene/gold plasmonic fiber-optic biosensor. Adv. Mater. Technol..

[231] Mishra A.K., Mishra S.K., Verma R.K. (2016). Graphene and beyond graphene MoS_2_: A new window in surface-plasmon-resonance-based fiber optic sensing. J. Phys. Chem. C.

[232] Yao B., Wu Y., Zhang A., Rao Y., Wang Z., Cheng Y., Gong Y., Zhang W., Chen Y., Chiang K. (2014). Graphene enhanced evanescent field in microfiber multimode interferometer for highly sensitive gas sensing. Opt. Express.

[233] Arasu P., Noor A., Shabaneh A., Yaacob M., Lim H., Mahdi M. (2016). Fiber Bragg grating assisted surface plasmon resonance sensor with graphene oxide sensing layer. Opt. Commun..

[234] Kaushik S., Tiwari U.K., Deep A., Sinha R.K. (2019). Two-dimensional transition metal dichalcogenides assisted biofunctionalized optical fiber SPR biosensor for efficient and rapid detection of bovine serum albumin. Sci. Rep..

[235] Hu H., Zavabeti A., Quan H., Zhu W., Wei H., Chen D., Ou J.Z. (2019). Recent advances in two-dimensional transition metal dichalcogenides for biological sensing. Biosens. Bioelectron..

[236] Yu D., Li J., Wang T., She X., Sun Y., Li J., Zhang L., Yu X.F., Yang D. (2020). Black phosphorus all-fiber sensor for highly responsive humidity detection. Phys. Status. Solidi Rapid Res. Lett..

[237] Hu Y., Huang Y., Tan C., Zhang X., Lu Q., Sindoro M., Huang X., Huang W., Wang L., Zhang H. (2017). Two-dimensional transition metal dichalcogenide nanomaterials for biosensing applications. Mater. Chem. Front..

[238] Tan S.F., Wu L., Yang J.K., Bai P., Bosman M., Nijhuis C.A. (2014). Quantum plasmon resonances controlled by molecular tunnel junctions. Science.

[239] Xu D., Xiong X., Wu L., Ren X.F., Png C.E., Guo G.C., Gong Q., Xiao Y.F. (2018). Quantum plasmonics: New opportunity in fundamental and applied photonics. Adv. Opt. Photonics.

[240] Fan W., Lawrie B.J., Pooser R.C. (2015). Quantum plasmonic sensing. Phys. Rev. A.

[241] Pooser R.C., Lawrie B. (2016). Plasmonic trace sensing below the photon shot noise limit. ACS Photonics.

[242] Lee C., Dieleman F., Lee J., Rockstuhl C., Maier S.A., Tame M. (2016). Quantum plasmonic sensing: Beyond the shot-noise and diffraction limit. ACS Photonics.

[243] Dowran M., Kumar A., Lawrie B.J., Pooser R.C., Marino A.M. (2018). Quantum-enhanced plasmonic sensing. Optica.

[244] McCormick C.F., Boyer V., Arimondo E., Lett P.D. (2007). Strong relative intensity squeezing by four-wave mixing in rubidium vapor. Opt. Lett..

[245] Lee J.S., Huynh T., Lee S.Y., Lee K.G., Lee J., Tame M., Rockstuhl C., Lee C. (2017). Quantum noise reduction in intensity-sensitive surface-plasmon-resonance sensors. Phys. Rev. A.

[246] Zhao Y., Peng Y., Hu X.G., Xia F., Zhao Q. (2020). Beating the shot-noise limit with optical fiber quantum sensors for salinity measurement. Sens. Actuators B Chem..

[247] Afek I., Ambar O., Silberberg Y. (2010). High-NOON states by mixing quantum and classical light. Science.

[248] Mitchell M.W., Lundeen J.S., Steinberg A.M. (2004). Super-resolving phase measurements with a multiphoton entangled state. Nature.

[249] Chen Y., Lee C., Lu L., Liu D., Wu Y.K., Feng L.T., Li M., Rockstuhl C., Guo G.P., Guo G.C. (2018). Quantum plasmonic N00N state in a silver nanowire and its use for quantum sensing. Optica.

[250] Zhou Z.K., Liu J., Bao Y., Wu L., Png C.E., Wang X.H., Qiu C.W. (2019). Quantum plasmonics get applied. Prog. Quant. Electron..

[251] Purcell E.M. (1995). Spontaneous emission probabilities at radio frequencies. Confined Electrons and Photons.

[252] Hakala T., Toppari J., Kuzyk A., Pettersson M., Tikkanen H., Kunttu H., Törmä P. (2009). Vacuum Rabi splitting and strong-coupling dynamics for surface-plasmon polaritons and rhodamine 6G molecules. Phys. Rev. Lett..

[253] Symonds C., Bonnand C., Plenet J., Bréhier A., Parashkov R., Lauret J., Deleporte E., Bellessa J. (2008). Particularities of surface plasmon–exciton strong coupling with large Rabi splitting. New J. Phys..

[254] Pelton M., Storm S.D., Leng H. (2019). Strong coupling of emitters to single plasmonic nanoparticles: Exciton-induced transparency and Rabi splitting. Nanoscale.

[255] Hensen M., Heilpern T., Gray S.K., Pfeiffer W. (2018). Strong coupling and entanglement of quantum emitters embedded in a nanoantenna-enhanced plasmonic cavity. ACS Photonics.

[256] Zhang J., Tang Y., Lee K., Ouyang M. (2010). Tailoring light–matter–spin interactions in colloidal hetero-nanostructures. Nature.

[257] Liu R., Zhou Z.K., Yu Y.C., Zhang T., Wang H., Liu G., Wei Y., Chen H., Wang X.H. (2017). Strong light-matter interactions in single open plasmonic nanocavities at the quantum optics limit. Phys. Rev. Lett..

[258] Santhosh K., Bitton O., Chuntonov L., Haran G. (2016). Vacuum Rabi splitting in a plasmonic cavity at the single quantum emitter limit. Nat. Commun..

[259] Chikkaraddy R., De Nijs B., Benz F., Barrow S.J., Scherman O.A., Rosta E., Demetriadou A., Fox P., Hess O., Baumberg J.J. (2016). Single-molecule strong coupling at room temperature in plasmonic nanocavities. Nature.

[260] Wen J., Wang H., Wang W., Deng Z., Zhuang C., Zhang Y., Liu F., She J., Chen J., Chen H. (2017). Room-temperature strong light–matter interaction with active control in single plasmonic nanorod coupled with two-dimensional atomic crystals. Nano Lett..

[261] Hatef A., Sadeghi S.M., Boulais É., Meunier M. (2012). Quantum dot–metallic nanorod sensors via exciton–plasmon interaction. Nanotechnology.

[262] Sadeghi S., Hatef A., Meunier M. (2013). Quantum detection and ranging using exciton-plasmon coupling in coherent nanoantennas. Appl. Phys. Lett..

[263] Qian Z., Ren J., Zhang F., Duan X., Gong Q., Gu Y. (2020). Nanoscale quantum plasmon sensing based on strong photon–exciton coupling. Nanotechnology.

[264] Kongsuwan N., Xiong X., Bai P., You J.B., Png C.E., Wu L., Hess O. (2019). Quantum plasmonic immunoassay sensing. Nano Lett..

[265] Ciracì C., Hill R., Mock J., Urzhumov Y., Fernández-Domínguez A., Maier S., Pendry J., Chilkoti A., Smith D. (2012). Probing the ultimate limits of plasmonic enhancement. Science.

[266] Savage K.J., Hawkeye M.M., Esteban R., Borisov A.G., Aizpurua J., Baumberg J.J. (2012). Revealing the quantum regime in tunnelling plasmonics. Nature.

[267] Scholl J.A., Koh A.L., Dionne J.A. (2012). Quantum plasmon resonances of individual metallic nanoparticles. Nature.

[268] Echarri A.R., Gonçalves P., Tserkezis C., de Abajo F.J.G., Mortensen N.A., Cox J.D. (2021). Optical response of noble metal nanostructures: Quantum surface effects in crystallographic facets. Optica.

[269] Oh S.H., Altug H., Jin X., Low T., Koester S.J., Ivanov A.P., Edel J.B., Avouris P., Strano M.S. (2021). Nanophotonic biosensors harnessing van der Waals materials. Nat. Commun..

[270] Zhu J., Ozdemir S.K., Xiao Y.F., Li L., He L., Chen D.R., Yang L. (2010). On-chip single nanoparticle detection and sizing by mode splitting in an ultrahigh-Q microresonator. Nat. Photonics.

[271] Liu W., Chen Y.L., Tang S.J., Vollmer F., Xiao Y.F. (2020). Nonlinear sensing with whispering-gallery mode microcavities: From label-free detection to spectral fingerprinting. Nano Lett..

[272] Yang D.Q., Chen J.H., Cao Q.T., Duan B., Chen H.J., Yu X.C., Xiao Y.F. (2021). Operando monitoring transition dynamics of responsive polymer using optofluidic microcavities. Light Sci. Appl..

[273] Quan Q., Burgess I.B., Tang S.K., Floyd D.L., Loncar M. (2011). High-Q, low index-contrast polymeric photonic crystal nanobeam cavities. Opt. Express.

[274] Yang D., Liu X., Li X., Duan B., Wang A., Xiao Y. (2021). Photoic crystal nanobeam cavity devices for on-chip integrated silicon photonics. J. Semicond..

[275] Baaske M.D., Foreman M.R., Vollmer F. (2014). Single-molecule nucleic acid interactions monitored on a label-free microcavity biosensor platform. Nat. Nanotechnol..

[276] Vincent S., Subramanian S., Vollmer F. (2020). Optoplasmonic characterisation of reversible disulfide interactions at single thiol sites in the attomolar regime. Nat. Commun..

[277] Jiao F., Li F., Shen J., Guan C., Khan S.A., Wang J., Yang Z., Zhu J. (2021). Wafer-scale flexible plasmonic metasurface with passivated aluminum nanopillars for high-sensitivity immunosensors. Sens. Actuat. B Chem..

[278] Knight M.W., King N.S., Liu L., Everitt H.O., Nordlander P., Halas N.J. (2014). Aluminum for plasmonics. ACS Nano.

[279] King N.S., Liu L., Yang X., Cerjan B., Everitt H.O., Nordlander P., Halas N.J. (2015). Fano resonant aluminum nanoclusters for plasmonic colorimetric sensing. ACS Nano.

[280] Wang Y., Yu J., Mao Y.F., Chen J., Wang S., Chen H.Z., Zhang Y., Wang S.Y., Chen X., Li T. (2020). Stable, high-performance sodium-based plasmonic devices in the near infrared. Nature.

[281] Yao Y., Liao Z., Liu Z.Q., Liu X., Zhou J., Liu G., Yi Z., Wang J. (2020). Recent progresses on metamaterials for optical absorption and sensing: A review. J. Phys. D Appl. Phys..

[282] Whitesides G.M., Grzybowski B. (2002). Self-assembly at all scales. Science.

[283] Li N., Tittl A., Yue S., Giessen H., Song C., Ding B., Liu N. (2014). DNA-assembled bimetallic plasmonic nanosensors. Light Sci. Appl..

[284] Lee S.W., Lee K.S., Ahn J., Lee J.J., Kim M.G., Shin Y.B. (2011). Highly sensitive biosensing using arrays of plasmonic Au nanodisks realized by nanoimprint lithography. ACS Nano.

[285] Klinkova A., Choueiri R.M., Kumacheva E. (2014). Self-assembled plasmonic nanostructures. Chem. Soc. Rev..

[286] Tittl A., Leitis A., Liu M., Yesilkoy F., Choi D.Y., Neshev D.N., Kivshar Y.S., Altug H. (2018). Imaging-based molecular barcoding with pixelated dielectric metasurfaces. Science.

[287] Yesilkoy F., Arvelo E.R., Jahani Y., Liu M., Tittl A., Cevher V., Kivshar Y., Altug H. (2019). Ultrasensitive hyperspectral imaging and biodetection enabled by dielectric metasurfaces. Nat. Photonics.

[288] Qin M., Sun M., Bai R., Mao Y., Qian X., Sikka D., Zhao Y., Qi H.J., Suo Z., He X. (2018). Bioinspired hydrogel interferometer for adaptive coloration and chemical sensing. Adv. Mater..

[289] He X., O’Keefe N., Liu Y., Sun D., Uddin H., Nirmalathas A., Unnithan R.R. (2018). Transmission enhancement in coaxial hole array based plasmonic color filter for image sensor applications. IEEE Photonics J..

[290] Lao J., Sun P., Liu F., Zhang X., Zhao C., Mai W., Guo T., Xiao G., Albert J. (2018). In situ plasmonic optical fiber detection of the state of charge of supercapacitors for renewable energy storage. Light Sci. Appl..

[291] Jin Y., Zhou L., Yu J., Liang J., Cai W., Zhang H., Zhu S., Zhu J. (2018). In operando plasmonic monitoring of electrochemical evolution of lithium metal. Proc. Natl. Acad. Sci. USA.

[292] Malkiel I., Mrejen M., Nagler A., Arieli U., Wolf L., Suchowski H. (2018). Plasmonic nanostructure design and characterization via deep learning. Light Sci. Appl..

[293] Li X., Shu J., Gu W., Gao L. (2019). Deep neural network for plasmonic sensor modeling. Opt. Mater. Express.

[294] Tittl A., John-Herpin A., Leitis A., Arvelo E.R., Altug H. (2019). Metasurface-based molecular biosensing aided by artificial intelligence. Angew. Chem. Int. Ed..

[295] Gomes J.C.M., Souza L.C., Oliveira L.C. (2021). SmartSPR sensor: Machine learning approaches to create intelligent surface plasmon based sensors. Biosens. Bioelectron..

